# Boosting singlet oxygen generation for salinity wastewater treatment through co-activation of oxygen and peroxymonosulfate in photoelectrochemical process

**DOI:** 10.1016/j.fmre.2022.12.007

**Published:** 2022-12-23

**Authors:** Qianqian Yang, Zhiyuan Feng, Yanbo Zhou, Hongying Zhao, Guohua Zhao

**Affiliations:** a Shanghai Key Lab of Chemical Assessment and Sustainability, Key Laboratory of Yangtze River Water Environment, School of Chemical Science and Engineering, Tongji University, Shanghai 200092, China; b Key Laboratory of Coal Gasification and Energy Chemical Engineering of Ministry of Education, East China University of Science and Technology, Shanghai 200092, China

**Keywords:** Photoelectrochemical process, Singlet oxygen, Oxygen activation, Peroxymonosulfate activation, Electron transfer, Saline wastewater

## Abstract

High concentrations of inorganic ions in saline wastewater pose adverse effects on hydroxyl radical (HO^•^)-dominated technologies. Here, we report a unique strategy for boosting singlet oxygen (^1^O_2_) generation via coactivation of oxygen and peroxymonosulfate (PMS) by regulating the electron transfer regime in the photoelectrochemical process. The Fe-N bridge in atomic Fe-modified graphitic carbon nitride (denoted SA-FeCN) favors the construction of electron-defective Fe and electron-rich N vacancies (Nvs) to accelerate directional electron transfer. The produced intermediate (HSO_4—_O···Fe−Nvs···O—O) as a chemical channel accelerates the directional electron transfer from PMS to further reduce O_2_ to form activated products (SO_5_^•−^, O_2_^•−^), thereby transforming O_2_ into ^1^O_2_. An optimized ^1^O_2_ generation rate of 39.4 μmol *L*^−^^1^*s*^−^^1^ is obtained, which is 15.7–945.0 times higher than that in traditional advanced oxidation processes. Fast kinetics are achieved for removing various phenolic pollutants in a nonradical oxidation pathway, which is less susceptible to the coexistence of natural organic matter and inorganic ions. The COD removal for coal wastewater and complex industrial wastewater in real scenarios is found to reach a value of 90%-96% in 3 h. This work provides a new direction for boosting the ^1^O_2_ generation rate, especially for the selective degradation of target electron-rich contaminants in saline wastewater.

## Graphical abstract

**Figure:**
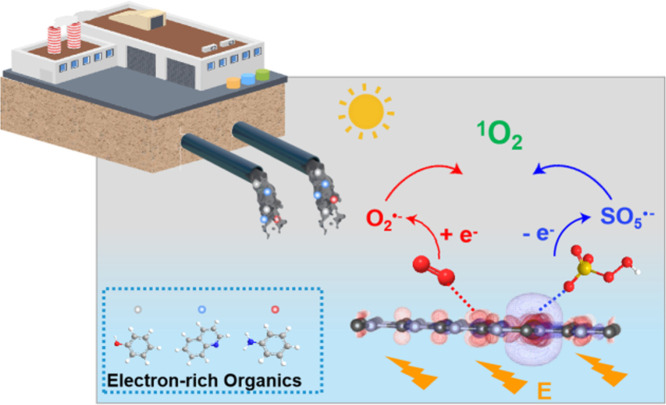

## 1 Introduction

High-salinity wastewater, characterized by high concentrations of soluble salts and refractory organic compounds, is generated in many industrial processes, including agro-food, tanning, and dyeing [1]. The discharge of such wastewater containing high salinity and organisms without prior treatment is known to adversely affect aquatic life, water portability and agriculture [2], [3], [4]. The integrated effects of salts and pollutants lead to serious inhibition of biological treatment [5]. In addition, existing inorganic ions (e.g., CO_3_^2−^, HCO_3_^−^and Cl^−^) can also pose adverse effects on the performance of SO_4_^•−^/HO^•−^dominated technologies, as they can quickly react with the high-powered oxidative species to generate byproducts [1,3]. Compared with SO_4_^•−^/HO^•^ radicals, ^1^O_2_, as a unique nonradical derivative of oxygen, is less susceptible to the water quality background (inorganic salt ions) [6,7]. In addition, ^1^O_2_ possesses an unoccupied π* orbital and exhibits high selectivity toward electron-rich organic pollutants such as medicine and pathogenic microorganisms in saline industrial wastewater [6,8].

The paramount advantages of ^1^O_2_ for wastewater treatment have already motivated increased research to boost its production. The main traditional methods include photosensitic reactions (e.g., molecular dyes or quantum nanodots for visible or UVA light adsorption) and enzymatic reactions (e.g., peroxidases or oxygenases for eliminating free radicals intracellularly) [6,9]. The current approach focuses on the activation of molecular oxygen (O_2_) and peroxymonosulfate (PMS). O_2_ is first reduced to superoxide anion radicals (O_2_^•−^) through a 1-electron reduction pathway, and then the generated O_2_^•−^ is simultaneously converted to ^1^O_2_ through recombination^6^, the Haber-Weiss reaction [10] and the oxidation reaction [11]. In addition, PMS is first oxidized to SO_5_^•−^ by the loss of the H atom, and then SO_5_^•‒^ rapidly self-reacts to generate ^1^O_2_ [7,12,13]_._ However, following pioneering reports, the conversion efficiency for O_2_ is greatly limited by the strict dynamics and thermodynamic barriers involved in the electron transfer processes [14]. Usually, less than 5% O_2_ in the medium can be catalytically transformed into reactive oxygen species (ROS) [15]. The PMS activation process requires the induction of a strong potential difference to fulfill the directional electron transfer from PMS to electron-poor regions [16]. In addition, whether in the O_2_ or PMS activation process, the formation of ^1^O_2_ is normally accompanied by the formation of other free radicals, such as HO^•^ and SO_4_^•‒^ [13,17,18]. In response to the deficiencies of traditional oxygen and/or PMS activation processes for ^1^O_2_ production, this work intends to provide insights into innovative solutions to increase the efficiency and selectivity for ^1^O_2_ generation.

The synergistic photoelectrocatalytic process has emerged as a promising and green alternative method since it can combine the advantages of electrocatalysis and photocatalysis [19]. It can overcome the energy barrier for forming intermediates in activation reactions with a high solar energy conversion efficiency, which is beneficial to improving the corresponding reactivity [11]. Specifically, photoelectrocatalytic activation promotes the formation of O_2_^•−^, which is the critical product for further oxidization to ^1^O_2_. Single atom catalysts (SACs) featuring high atom utilization efficiency and tunable electronic structure show high selectivity and activity in PMS and O_2_ catalytic reactions [20], [21], [22]. Many previous studies suggest that vacancies can enrich local electrons and inhibit the recombination of photogenerated electrons and holes [23], [24], [25]. Hence, surface vacancies could serve as electron donation sites to enhance the performance of O_2_ activation [25]. Very recently, SACs with a FeN_4_ structure have been widely employed to generate ROS for environmental remediation [12,13]. Moreover, uneven charge distribution caused by atomic Fe sites can be used to construct electron-rich and electron-deficient areas [12]. Graphitic carbon nitride (CN) has a high N content and specifies C and N sites, resulting in abundant and well-distributed single atomic fixation sites [13,26,27]. Moreover, CN has been demonstrated as a very promising solar-energy-driven metal-free photocatalyst for realizing desirable electronic band structures and high physicochemical stability [28], [29], [30].

Herein, in this work, we propose a novel strategy to boost both the activity and selectivity for ^1^O_2_ generation in the synergistic photoelectrocatalytic activation of oxygen and PMS. Single-atom Fe anchored with N sites and N vacancies was fabricated on graphitic carbon nitride and applied as a photoelectrocathode (denoted SA-FeCN). The interaction through the Fe-N bridge favors the construction of electron-defective Fe and electron-rich N vacancy centers for accelerating directional electron transfer in the activation process. A unique synergistic activation mechanism for PMS oxidation on electron-deficient sites along with O_2_ reduction on electron-rich sites is first proposed. The complete removal of 3-chlorophenol (3-CP) is achieved in 6 min with the SA-FeCN electrode, and the optimized apparent rate constant (*k*_obs_) can reach as high as 0.57 min^−1^. In addition, the COD removal for high salinity wastewater in real scenarios reaches a value of 90%−96% in 3 h, confirming that metal stable ^1^O_2_ is efficient for the purification of high salinity wastewater containing electron-rich contaminants.

## 2 Experimental section

### 2.1 Materials

Melamine (MA, C_3_H_6_N_6_, 99%, CAS: 108–78–1) and cyanuric acid (CA, C_3_H_3_N_3_O_3_, 99%, CAS: 108–80–5), carbon paper (CP), 3-chlorophenol (3-CP, 98%, CAS: 108–43–0), 2,4-dichlorophenol (DCP, >99.5%, CAS: 120–83–2), 2,4,5-trichlorophenol (2,4,5-TCP, >95%, CAS: 95–95–4), p-nitrophenol (4-NP, >99%, CAS:100–02–7), sodium sulfate (Na_2_SO_4_, >99.0%, CAS: 7757–82–6), sodium chloride (NaCl, >99.0%, CAS: 7647–14–5), furfuryl alcohol (FFA, >98.0%, CAS: 98–00–0), deuteroxide (D_2_O, >98%, CAS: 7789–20–0), dimethylpyridine N-oxide (DMPO, >98.0%, CAS: 1073–23–0), 2,2,6,6-tetramethyl-4-piperidinol (TEMP, >98.0%, CAS: 2403–88–5) were bought from Aladdin Bio-Chem Technology Co., Ltd (Shanghai, China). Peroxymonosulfate (PMS, active oxygen >4.5%, CAS: 37,222–66–5), sodium hydroxide (KOH, >95%, CAS: 1310–58–3), acetone(98%, CAS: 15,364–56–4), methanol(>99.9%, CAS: 67–56–21), and H_2_SO_4_ (AR, 98%, CAS: 664–93–9), sodium bicarbonate (NaHCO_3_, >99.0%, CAS: 144–55–8), sodium fluoride (NaF, >98.0%, CAS: 7681–49–4), magnesium sulfate (MgSO_4_, >99.0%, CAS: 7487–88–9), calcium sulfate (CaSO_4_, AR, >99.0%, CAS: 7778–18–9), acetonitrile (>99.5%, CAS: 75–05–8) were purchased from Sinopharm Chemical Reagent Co., Ltd (Shanghai, China). Superoxide Dismutase (SOD, >1400 units/mg, 9054–89–1) and anthracene-9,10-dipropionic acid disodium salt (ADPA, >98%, CAS: 82,767–90–6) were obtained from Meryer (Shanghai) Chemical Technology Co., Ltd. Standard Suwannee River NOM (SR-NOM) obtained from the International Humic Substances Society was used as a model NOM to investigate the interference of possible co-existed NOM on pollutant degradation. Oxygen (O_2,_ 99.9%, CAS: 7782–44–7), argon (Ar, 99.9%, CAS: 7440–37–1) and nitrogen (N_2_, 99.9%, CAS: 7727–37–9) were purchased from Shanghai Qingkuan Chemical Co., Ltd. (Shanghai, China). All chemical reagents used in this research were of analytical grade and could be used without any further purification. In addition, deionized water (DI) has been used throughout the experiments.

### 2.2 Synthesis of SA-FeCN and NP-FeCN catalysts

Melamine (3 g) and cyanuric acid (2 g) were dissolved and stirred in 80 mL ethanol at 70 °C for 30 min to obtain solution A. Fe(Cl)_3_•6H_2_O (100, 200, 300 mg) and oxalic acid dihydrate (100 mg) were dissolved in 50 mL of deionized water with vigorous magnetic stirring at room temperature for 30 min to obtain solution B. Next, solution A was added to solution B to obtain mixed solution C. Solution C was continuously stirred at 85 °C until the solvent was completely evaporated. The supermolecule precursor was collected and dried at 60 °C overnight. The obtained mixture was ground and heated to 520 °C at a rate of 2.5 °C min^−1^ under an argon atmosphere and kept for 4 h. Then, the as-obtained powder was further heated at 620 °C for 2 h under argon atmosphere protection at a rate of 5 °C min^−1^. Finally, the SA-FeCN catalyst was obtained after treatment in 0.1 mol *L*^−^^1^ H_2_SO_4_ at 60 °C for 3 h to thoroughly eliminate unstable metal content species. NP-FeCN was obtained by heating a mixture of iron particles and melamine at 550 °C for 4 h. The preparation of SA-FeCN/CP and NP-FeCN/CP cathodes is described in detail in the supporting information. Finally, the iron content in SA-FeCN/CP and NP-FeCN/CP was determined to be 0.36 wt% by ICP analysis.

### 2.3 Characterization methods

The crystal configuration of the catalysts was probed by X-ray diffraction (XRD), and the patterns were collected on Rigaku-D/max2550 powder diffractometer operated at 40 kV and 30 mA with Cu Kα as irradiation (λ = 0.15405 nm). The morphology and microstructure of catalysts were characterized by transmission electron microscopy (TEM), the images were obtained through a JEM-2010F electron microscope. High-angle annular dark-field (HAADF) images were acquired with a JEM-ARM200F TEM/STEM with a spherical aberration corrector working at 300 kV. Elemental analysis (EA) was measured on a vario MICRO CUBE elementar. Inductively coupled plasmaatomic emission spectrometry (ICP-AES) was introduced to determine the content of doped metal in synthesized samples and metal leaching conditions during electrocatalytic degradation process using PE OPTIMA 2100DV. The UV–Vis diffuse reflectance spectra (DRS) were collected on Agilent Carry5000 using BaSO_4_ as reference material. The photoluminescence (PL) spectra were recorded on a Hitachi F-7000 spectrometer at room temperature. Fourier transform infrared (FTIR) spectra were carried out on an IR Vertex 70 FTIR spectrometer. The electron paramagnetic resonance (EPR) signals of the reactive species produced in the reaction were captured by DMPO and TEMP, and detected on a Bruker EMX X plus-10/12 ESR spectrometer. Total organic carbon (TOC), dissolved organic carbon (DOC) and dissolved organic nitrogen (DON) were analyzed through a TOC analyzer (multi N/C 3100 TOC/TN analyzer, Analytikjena, Germany). The chemical oxygen demand (COD) was obtained from HACH DR3900 instrument. What's more, the X-ray absorption fine structure (XAFS) spectroscopy data were obtained at BL11B and BL08U1A beamline in Shanghai Synchrotron Radiation Facility (SSRF). All the obtained data are expressed as mean ± standard deviation (μ ± σ). The calculation formula of standard deviation (σ) is shown as Eq. 1:

(1) $$\sigma =\sqrt{\frac{\sum_{i=1}^{n}{\left(x_{i}-\mu \right)}^{2}}{n}}$$

where σ is standard deviation (s.d.), x_i_ is the actual measured value, μ is the mean value, n refers to the number of repeated experiments.

### 2.4 Degradation procedure

The degradation experiment for organic contaminants in the photoelectrocatalytic reaction was performed in a single-cell quartz tubular reactor with a circulating cooling system to keep the reactor at a constant temperature of 25 °C. The obtained SA-FeCN and NP-FeCN was used as photocathodes, while Pt foil was used as the anode. Sunlight was simulated by a 300 W xenon lamp (PLS-SXE300D) fitted with a filter to eliminate UV irradiation (λ < 420 nm). A 30 mL solution of 10 ppm organic compounds (3-CP, 2,4-DCP, 2,4,6-DCP and 4-NP) with PMS (0.005 M) at pH 7 was used as simulated wastewater. The degradation experiment was carried out at a constant current of 0.02 A under an O_2_-saturated solution, and the average potential for SA-FeCN and NP-FeCN was 2.8 V and 3.1 V, respectively. The stability experiments were carried out 10 times as follows: the recyclability degradation experiments were revealed by collecting the photocathode, washing with deionized water, drying under a vacuum oven, and applying it for another reaction under the same experimental conditions.

### 2.5 Analytical procedure

High-performance liquid chromatography (HPLC, Agilent 1260 Infinity, Germany) was used to record the concentration of pollutants during the degradation process. The mobile phases and detection wavelengths were set as follows: methanol/water (70:30, v/v) with λ = 273 nm for 3-CP, methanol/water (30:70, v/v) with λ = 254 nm for 2,4-DCP, acetonitrile/water (35:65, v/v) with λ = 266 nm for 2,4,5-TCP, acetonitrile/water (60:40, v/v) with λ = 220 nm for 4-NP. In all the above HPLC processes, the column temperature was maintained at 25 °C, and the flow rate was 1 ml·min^−1^.

## 3 Results and discussion

### 3.1 Intrinsic surface properties of atomic iron active sites in photoelectrodes

**Fig. 1:**
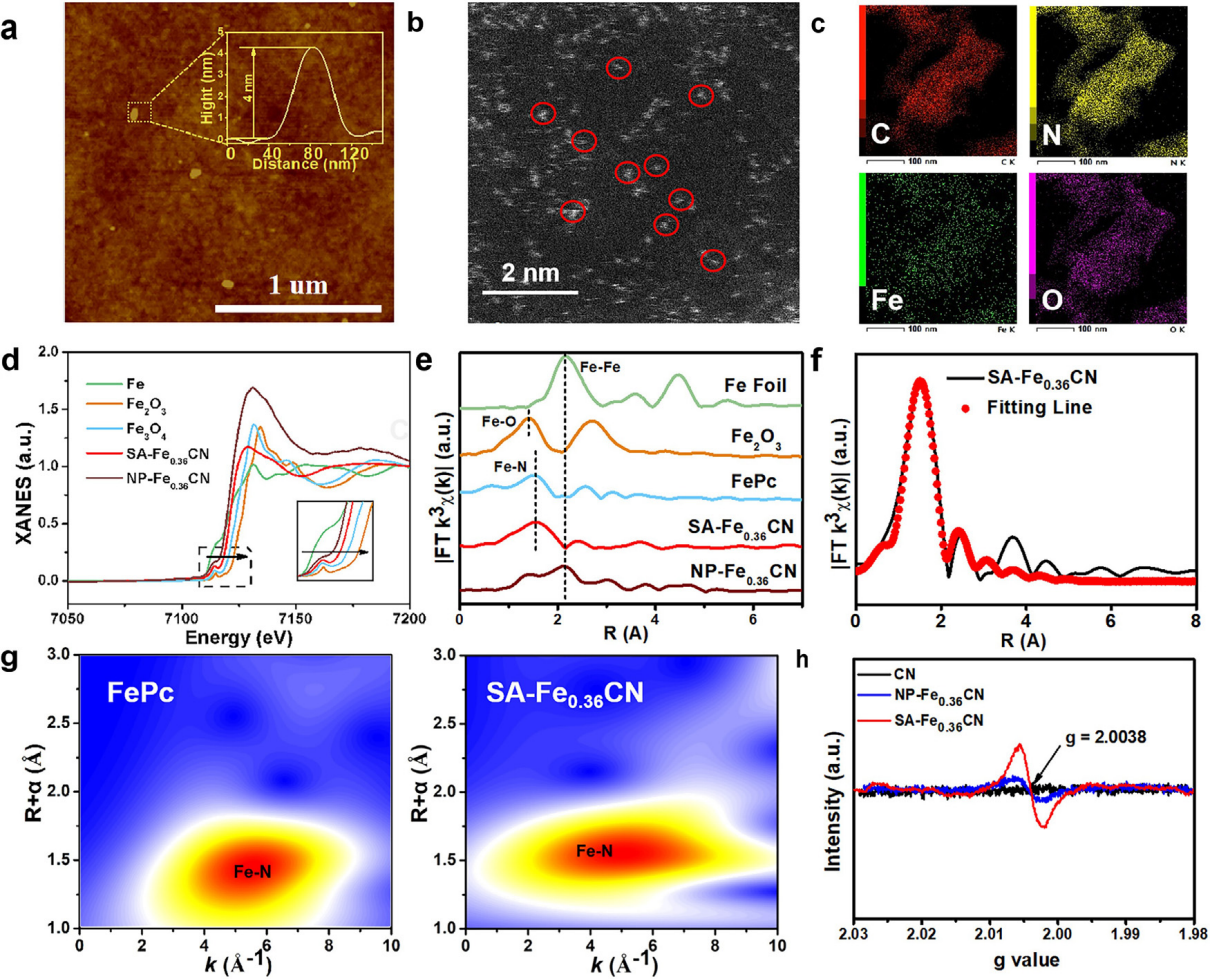
(a) AFM image of SA-Fe_0.36_CN. (b) Atomic-resolution HAADF-STEM image of SA-Fe_0.36_CN. (c) EDX images of the SA-Fe_0.36_CN. (d) Fe K-edge XANES and (e) k^3^-weighted Fourier-transformed curve of Fe foil, FePc, Fe_2_O_3_, NP-Fe_0.36_CN and SA-Fe_0.36_CN. (f) EXAFS R space fitting result for SA-Fe_0.36_CN. (g) WT-EXAFS for FePc and SA-Fe_0.36_CN, respectively. (h) Room temperature EPR spectra of CN, SA-Fe_0.36_CN and NP-Fe_0.36_CN.

Atomic force microscopy (AFM) imaging reveals an ultra thin SA-Fe_0.36_CN nanosheet with a thickness of approximately 4 nm, corresponding to approximately six atomic layers (Fig. 1a). To discern the isolated iron atoms in the SA-Fe_0.36_CN photoelectrode, high-angle annular dark field scanning transmission electron microscopy (HAADF-STEM) was performed. All the iron atoms (small bright dots circled in red) were atomically dispersed over the entire CN matrix (Fig. 1b). The element mapping further confirmed that atomic ions are uniformly distributed on CN together with a coverage of O (Fig. 1c). To explore the coordination environment and chemical state of Fe sites, X-ray absorption near-edge structure (XANES) spectroscopy was carried out. The position of the rising edge for SA-Fe_0.36_CN is found to be situated between that for Fe foil and Fe_2_O_3_, suggesting that a single Fe atom carries a positive charge and that the average oxidation state of Fe ranges between Fe^0^ and Fe^3+^ (Fig. 1d). Meanwhile, the valence of SA-Fe_0.36_CN was calculated to be + 2.37 from the linear fitting curve shown in Figure S10. The Fourier transformed k^3^-weighted extended X-ray absorption fine structure (FT-EXAFS) spectrum (Fig. 1e) for SA-Fe_0.36_CN demonstrates a solitary peak at approximately 1.6 Å, which is ascribed to the Fe-N bond [13,31,32]. Compared with Fe foil, Fe_2_O_3_, no Fe-O or Fe-Fe peaks are observed, verifying the absence of Fe nanoclusters and/or oxides in SA-Fe_0.36_CN. The EXAFS fitting curve reveals that the coordination number of Fe is 4. All the above results indicate that the Fe atoms in SA-Fe_0.36_CN are dispersed at the atomic level with a Fe-N_4_ configuration (Fig. 1e). In the wavelet transform (WT) contour plots (Fig. 1g), the SA-Fe_0.36_CA exhibits the same intensity maximum with FePc at ∼5.0 *Å*^−1^, which could be assigning to Fe-N_4_ coordination, confirm that the Fe atoms in Fe-N_4_ are atomically dispersed with a Fe-N_4_ configuration in SA-Fe_0.36_CA. [24,33]. Moreover, as revealed in Fig. 1h, SA-Fe_0.36_CN exhibits a distinct EPR signal at *g* = 2.0038, which can be ascribed to the electrons trapped in the nitrogen vacancies on the surface of the nanosheets [24,34]. In accordance with element analysis (EA), the C to N ratio for SA-Fe_0.36_CN is determined to be 0.81:1 (Table S2). The above results confirm the presence of single-atom Fe and abundant nitrogen vacancies in the synthesized SA-Fe_0.36_CN.

### 3.2 The mechanism insight of coactivation for oxygen and PMS in photoelectrochemical process

**Fig. 2:**
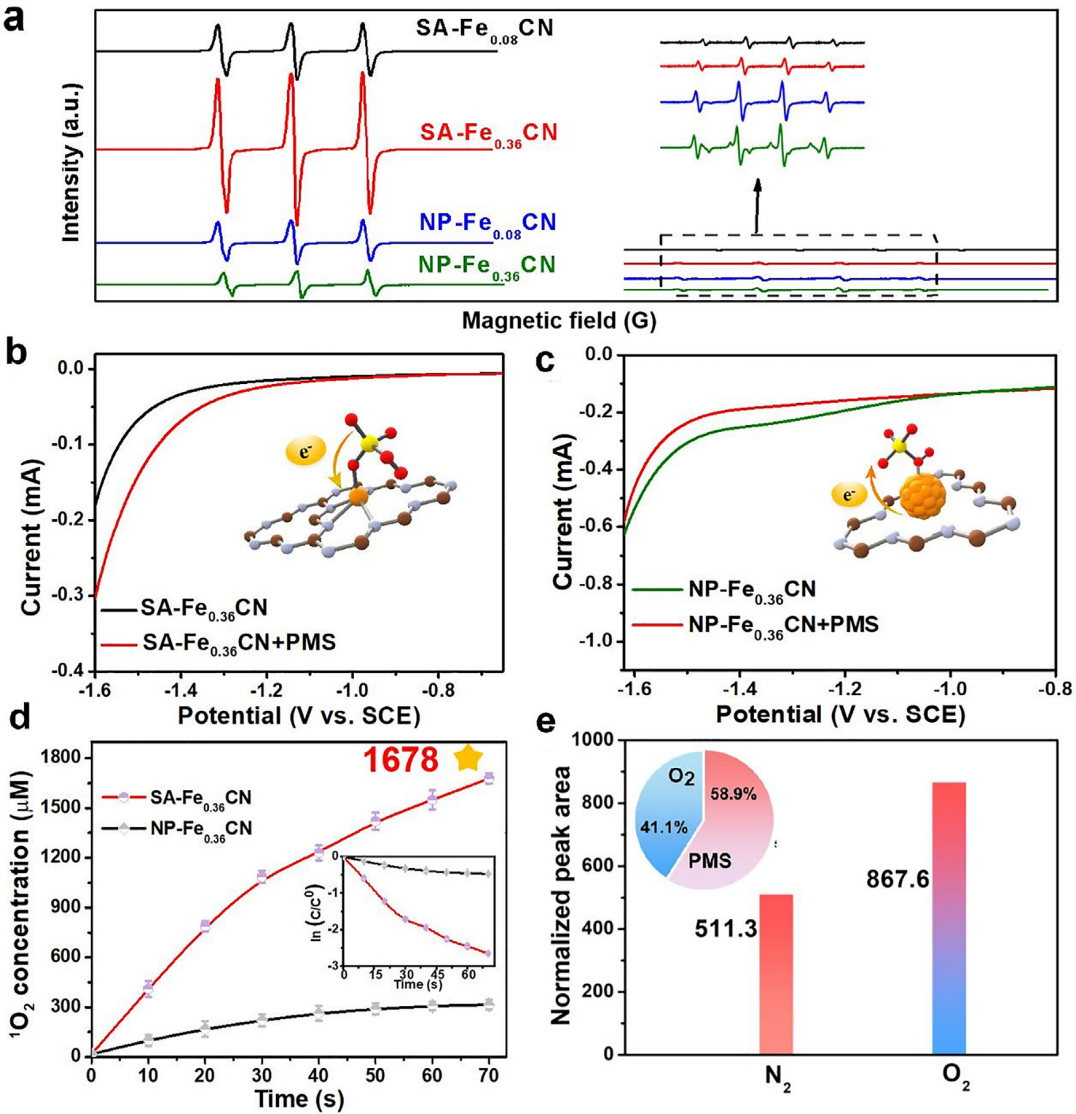
(a) EPR spectra of TEMP-^1^O_2_, DMPO-SO_4_^•−^, DMPO—HO^•^ adduct for different catalyst in photoelectrochemical PMS and oxygen co-activation process. (b) LSV curves of SA-Fe_0.36_CN. (c) LSV curves of NP-Fe_0.36_CN. (d) Cumulative ^1^O_2_ concentration of SA-Fe_0.36_CN and NP-Fe_0.36_CN in photoelectrochemical PMS and oxygen co-activation process, (e) TEMP-^1^O_2_ normalized peak area of SA-Fe_0.36_CN under N_2_ and O_2_ in presence of PMS.

To identify the active radicals formed during the synergic PMS and O_2_ activation process, the electron spin resonance (EPR) technique was employed to directly detect the reactive oxygen species (ROS) [35,36]. By increasing the concentration of atomic Fe sites in the SA-Fe_0.36_CN electrodes, the generation of ^1^O_2_ is obviously enhanced but not for HO^•^. For NP-Fe_0.36_CN, the formation of both ^1^O_2_ and HO^•^ is almost independent of the concentration of doped Fe. In addition, the intensity of the triplet signals for TEMP-^1^O_2_ with SA-Fe_0.36_CN is 5 times higher than that for NP-Fe_0.36_CN (Fig. 2a). The overwhelming majority of ^1^O_2_ suggests a synergetic promotion effect in the SA-Fe_0.36_CN/PMS+O_2_ system. Based on the possible reactions that can occur during PMS and O_2_ activation processes, PMS can be oxidized to PMS anion radical (SO_5_^•−^) by atomic Fe as an electron accepter in SA-Fe_0.36_CN (Eq. 2). Then, the self-reaction of SO_5_^•−^ leads to ^1^O_2_ generation (Eqs. 3-4) due to the high reaction rate (≈ 2 × 10^8^ *M* ^−^^1^ *s* ^−^^1^) and low activation energy (7.4 ± 2.4 kcal mol^−1^) [13,22]. In addition to PMS activation, the superoxide anion radical (O_2_^•−^) as an intermediate in the stepwise reduction of oxygen is critical for ^1^O_2_ synthesis (Eq. 5). The constructed chemical channel (HSO_4—_O···Fe−Nvs···O—O, verified by the following DFT investigation) accelerates the directional electron transfer from PMS to further reduce O_2_ to form activated products (SO_5_^•−^, O_2_^•−^). Benefiting from the continuous formation of O_2_^•‒^ in the photoelectrochemical process, ^1^O_2_ can be triggered through the recombination of O_2_^•−^/O_2_^•−^ (Eq. 6). Although the Haber-Weiss reaction can also be feasible for ^1^O_2_ generation, this pathway is negligible in this work due to the very limited amount of HO^•^ and H_2_O_2_ present (Fig. S12).

(2) $$HSO_{5}^{-}\overset{Fe-N}{\to }SO_{5}^{•-}+H^{+}+e_{PMS}^{-}$$

(3) $$SO_{5}^{•-}+SO_{5}^{•-}\to {}^{1}O_{2}+2SO_{4}^{2-}$$

(4) $$SO_{5}^{•-}+SO_{5}^{•-}\to {}^{1}O_{2}+S_{2}O_{8}^{2-}$$

(5) $$O_{2}+e_{cb}^{-}/e\to O_{2}^{•-}$$

(6) $$H^{+}{+2O}_{2}^{•-}\to {}^{1}O_{2}+HO_{2}^{-}$$

To confirm the electron transfer pathway between PMS and atomic Fe in the SA-Fe_0.36_CN electrode, linear sweep voltammetry (LSV) analyses were carried out. The current density with the SA-Fe_0.36_CN electrode is significantly increased following the introduction of PMS, indicating that the electrons in PMS are transferred to single Fe atoms (Fig. 2b). In contrast, the current density with the NP-Fe_0.36_CN electrode is decreased due to the electron transfer from NP-Fe_0.36_CN to PMS (Fig. 2c). These results confirm that the electron transfer pathways in the SA-Fe_0.36_CN/PMS and NP-Fe_0.36_CN/PMS systems are completely opposite.

The cumulative ^1^O_2_ concentration with SA-Fe_0.36_CN was further quantified by the methyl blue (MB)-anthracene-9,10-dipropionic acid disodium salt (ADPA) method [37] (Text S2). As shown in Fig. 2d, the concentration of ^1^O_2_ is rapidly increased to 1678 μmol *L*^−^^1^ with increasing photoelectrocatalysis reaction time in 70 s, which is 5.3 times higher than that obtained for NP-Fe_0.36_CN (315 μmol *L*^−^^1^) under the same conditions. In addition, the ^1^O_2_ generation rate in this work can reach a value of up to 39.4 μ mol *L*^−^^1^*s*^−^^1^, which is much higher than that obtained for traditional H_2_O_2_ activation, sole PMS activation and O_2_ activation processes (Table S3). To distinguish the contribution of PMS and O_2_ activation to the generation of ^1^O_2_, the normalized double integration of the obtained EPR spectra was conducted to semiquantify the ^1^O_2_ concentration using MATLAB software (Figs. S15-S16, Text S1) [10]. Based on the above summarized activation reactions shown in Eqs. 2-6, the ^1^O_2_ generated under a N_2_ atmosphere can be assigned to PMS activation. As expected, the TEMP-^1^O_2_ normalized peak areas for SA-Fe_0.36_CN under O_2_ and N_2_ atmospheres are 867.6 and 511.3, respectively, indicating that 58.9% of ^1^O_2_ generation is contributed by PMS activation (Fig. 2e).

### 3.3 Theoretical investigation of coactivation for oxygen and PMS in photoelectrochemical process

**Fig. 3:**
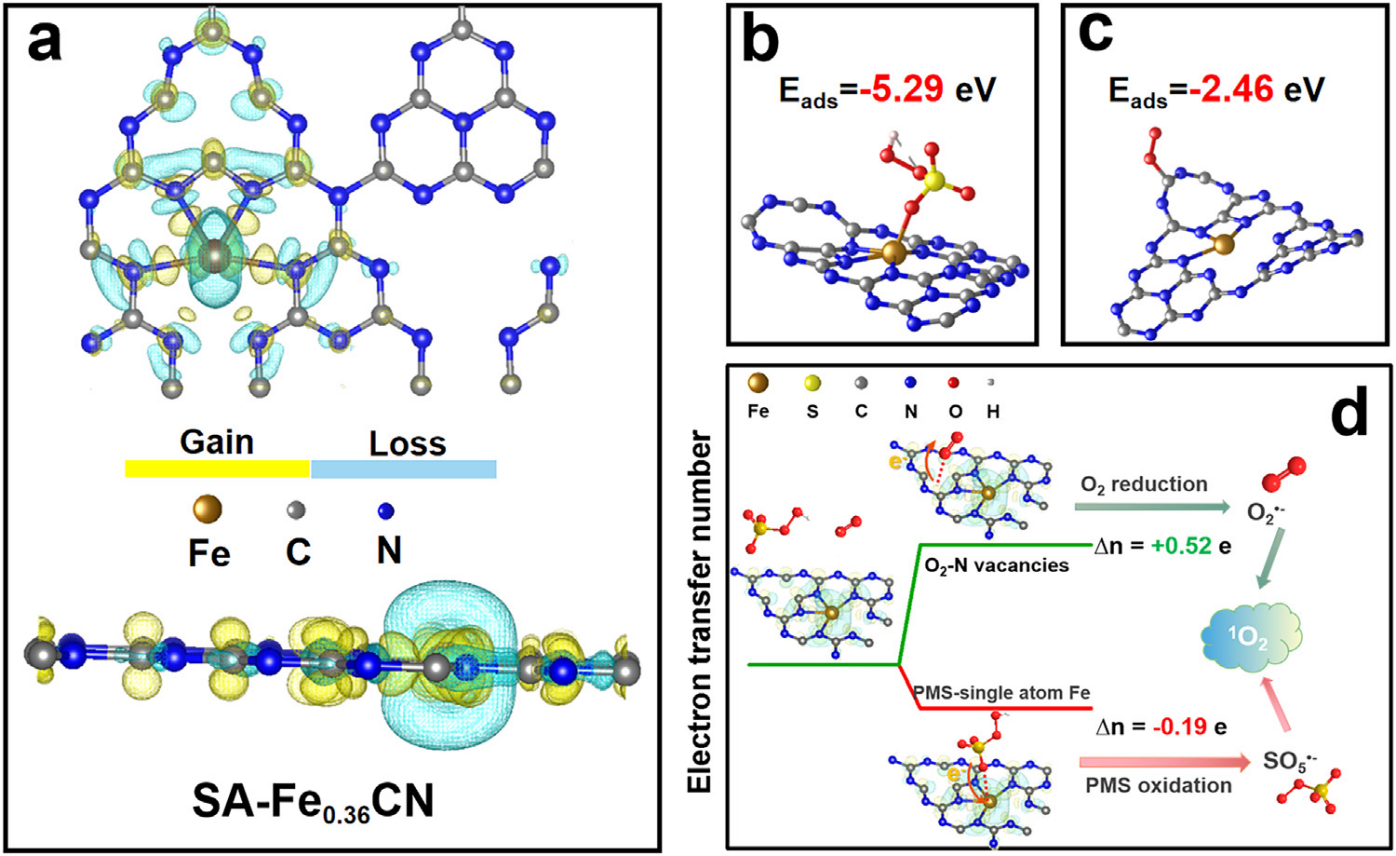
(a) Top view and side view of the charge density difference of SA-Fe_0.36_CN; (b) The adsorption energy after PMS adsorption on the surface of SA-Fe_0.36_CN; (c) The adsorption energy after O_2_ adsorption on the surface of SA-Fe_0.36_CN; (d) The electron transfer orientation and number of co-activation PMS and O_2_ process.

To deeply understand the coactivation mechanism with O_2_ and PMS in the photoelectrochemical process, density functional theory (DFT) calculations were undertaken to probe the charge distribution and electron transfer for the SA-Fe_0.36_CN electrode. The charge density difference analysis for the formation of single atom Fe and N vacancies (Nvs) is shown in Fig. 3a. The cyan region represents charge depletion, and the yellow region indicates charge accumulation. It can be observed that the charge around the single-atom Fe is lost, indicating the formation of an electron-defective region, while Nvs accumulate most of the negative electrons to form an electron-rich region. In addition, the Nvs can serve as electron trap states to accumulate photoelectrons under light illumination. Therefore, we propose that PMS can adsorb onto single-atom Fe and donate electrons to electron-defective Fe, and O_2_ obtains electrons at electron-rich Nvs.

The adsorption energy of PMS on single-atom Fe (−5.29 eV) and Nvs (3.26 eV) further demonstrated that PMS can only be activated on single-atom Fe, while adsorption on Nvs is thermodynamically impossible (Fig. 3b; Table S5). However, there is little difference in the adsorption energy of O_2_ on single-atom Fe (−2.74 eV) and Nvs (−2.46 eV, Fig. 3c; Table S5). Due to the steric hindrance effect, O_2_ can only adsorb onto the Nvs. Hence, based on the above DFT calculation results, we propose that the reaction intermediate HSO_4—_O···Fe−Nvs···O—O will be formed during the coactivation of O_2_ and PMS. The adsorbed PMS donates −0.19 e to single-atom Fe and then tends to be oxidized to SO_5_^•−^ (see Table S6 for details). Meanwhile, it should be noted that the Nvs can trap one O atom of O_2_ to achieve electron-oriented transfer from Nvs to O_2_ (0.52 e). Hence, the above discussion indicates that the produced intermediate (HSO_4—_O···Fe−Nvs···O—O) can serve as a chemical channel facilitating directional electron transfer from PMS to further reduce O_2_ to form activated products (SO_5_^•−^, O_2_^•−^), thereby further boosting ^1^O_2_ production, which coincides with the experimental result (Fig. 3d).

### 3.4 The evaluation of electron-rich organic pollutants removal with ^1^O_2_ in photoelectrochemical process

**Fig. 4:**
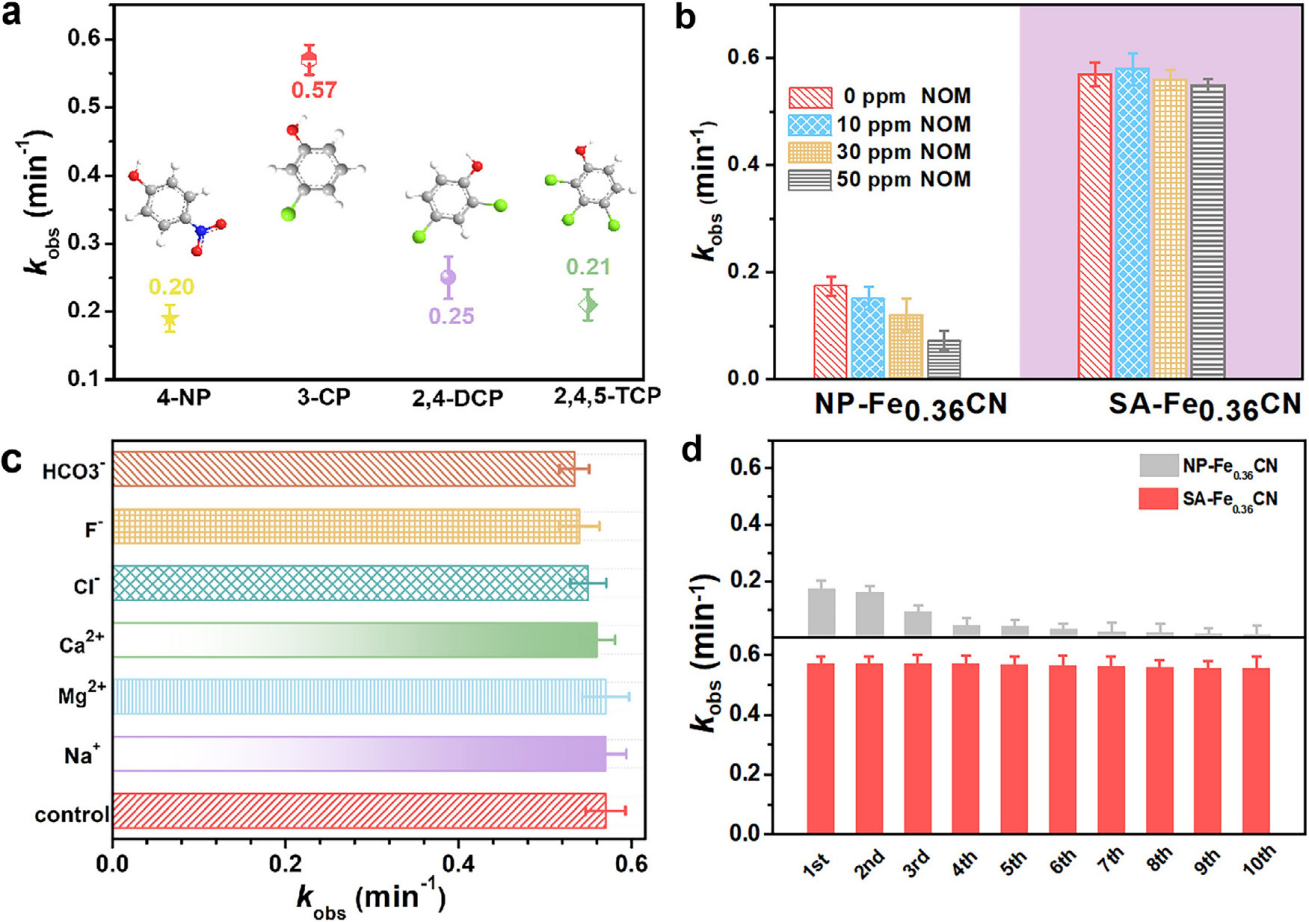
(a) Degradation rate of 4-NP, 3-CP, 2,4-DCP, 2,4,5-TCP by SA-Fe_0.36_CN co-activated PMS and O_2_; (b) The effect of NOM on the degradation rate of 3-CP with SA-Fe_0.36_CN and NP-Fe_0.36_CN; (c) The effect of different ions on degradation efficiency of 3-CP with SA-Fe_0.36_CN; (d) The cycle stability experiments with SA-Fe_0.36_CN and NP-Fe_0.36_CN.

The optimized generation of ^1^O_2_ with the SA-Fe_0.36_CN photoelectroelectrode through synergetic PMS and O_2_ activation was further evaluated by eliminating electron-rich organic pollutants. As shown in Fig. 4a, excellent removal performance toward phenolic pollutants is observed. In particular, the apparent rate constant (*k*_obs_) for 3-CP can reach a value of 0.57 min^−1^, which is 3.4 times higher than that for NP-Fe_0.36_CN. This observation confirms that ^1^O_2_, as an electrophilic species, is prone to attacking sites with electron-donating groups [38]. Natural organic matter (NOM) is a complex mixture of organic substances that is extensively distributed in ground water, surface water and wastewater [30,39,40]. Hence, different concentrations of NOM are used to evaluate the degradation performance of the SA-Fe_0.36_CN/PE(PMS/O_2_) system during actual wastewater treatment. As expected, the degradation rate of 3-CP is maintained at a high level (0.550 min^−1^) even with increasing NOM concentration to 50 mg·*L*^−^^1^ for SA-Fe_0.36_CN (Fig. 4b). However, the *k*_obs_ for 3-CP with NP-Fe_0.36_CN declines by 59% from 0.174 to 0.072 min^−1^. This observation implies the high selectivity of the SA-Fe_0.36_CN/PE(PMS/O_2_) process for eliminating electron-rich organic pollutants over coexisting NOM. Additionally, inorganic ions unavoidably exist in real water-treatment scenarios, which can have a negative effect on the degradation of organics [1,40,41]. Various ions, including HCO_3_^−^, *F*^−^, Cl^−^, Ca^2+^, Mg^2+^, and Na^+^, exhibit negligible effects on the degradation efficiency for 3-CP in the SA-Fe_0.36_CN/PE(PMS/O_2_) system (Fig. 4c), further demonstrating a nonradical oxidation mechanism. However, the *k*_obs_ for 3-CP is decreased from 0.174 min^−1^ to 0.122 min^−1^ in the presence of *F*^−^ in the NP-Fe_0.36_CN/PE(PMS/O_2_) system (Fig. S22). This is can be attributed to the formation of the Fe(III)-F complex, suppressing the interaction of PMS with Fe^0^ or Fe_2_O_3_ to form HO^•^. The degradation efficiency with the SA-Fe_0.36_CN electrode remains almost as high as 0.556 min^−1^ after 10 successive runs, leading to a loss of only 2.5% compared to the first run (0.570 min^−1^), and trace Fe leaching (< 0.01 ppm) is achieved during the degradation process. However, an apparent decline (93.1%) occurs with NP-Fe_0.36_CN after 10 cycles, possibly due to the continuous leaching of Fe^0^ and Fe_2_O_3_ nanoparticles during the reaction (Fig. 4d).

**Fig. 5:**
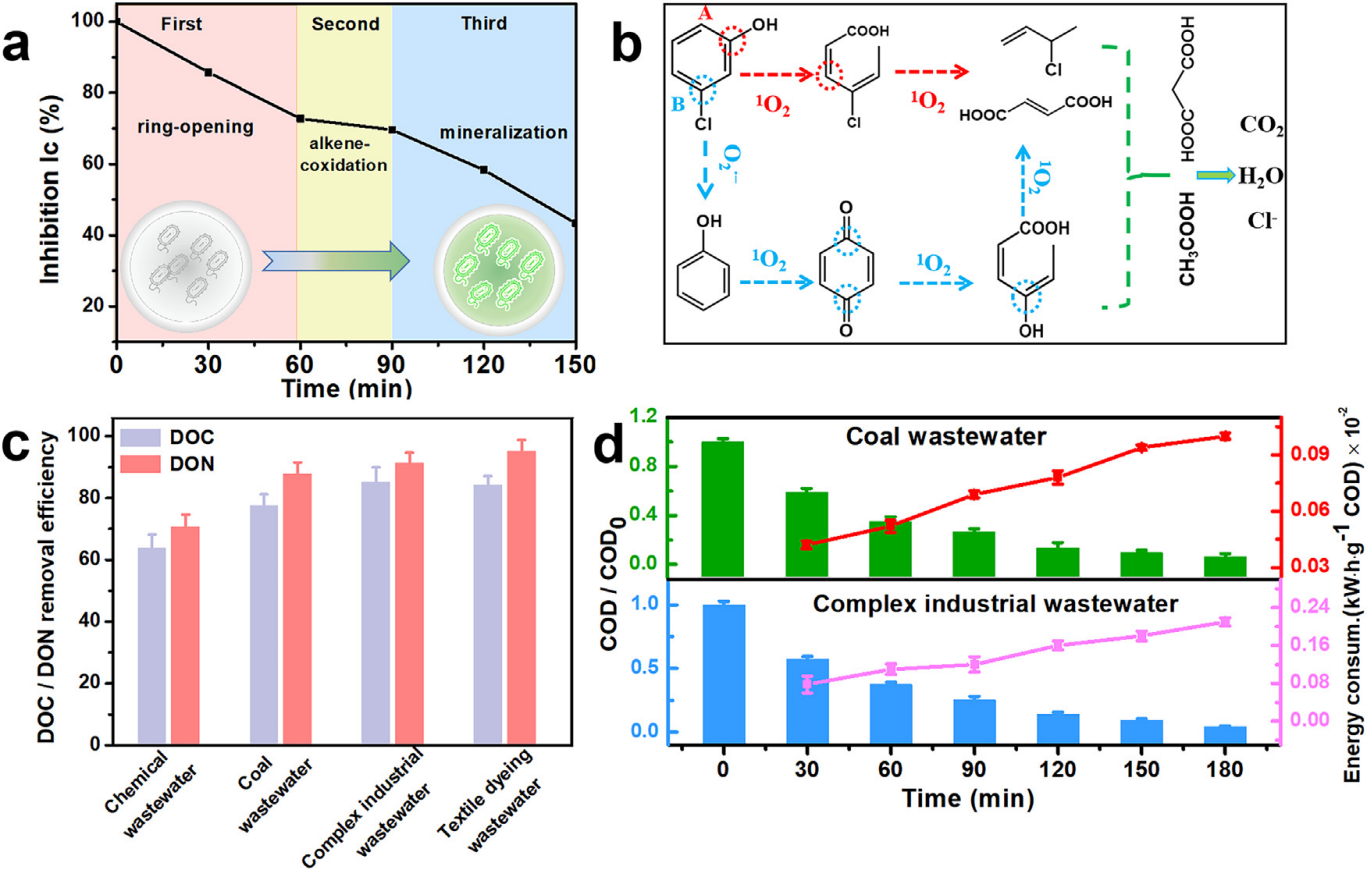
(a) Toxicity evolution of 3-CP degradation process with SA-Fe_0.36_CN; (b) The degradation pathway of 3-CP; (c) DOC/DON removal efficiency of different high salinity wastewater after 4 h degradation; (d) The COD removal efficiency and energy consumption for the wastewater treatment.

Chlorophenols (CPs) are widely applied in the petrochemical and agricultural industries and show a high bioaccumulation ability, strong cytotoxicity, and carcinogenicity [35]. Furthermore, some CP degradation intermediates are more toxic [21]. Hence, for potential application in environmental cleanup, the possible toxicity of 3-CP degradation intermediates was examined based on the inhibition of the marine bacterium Brio fischeri [36]. In detail, the corresponding degradation intermediates for 3-CP are summarized in Table S8. As shown in Fig. 5a, the luminescence inhibition rate is decreased from 100% to 43.3% after 150 min, reflecting the obviously decreased acute toxicity. Combined with the proposed degradation pathway shown in Fig. 5b, the reduced toxicity can be divided into three intervals. First, in pathway A, the hydroxyl functional group of the aromatic ring in 3-CP is attacked by ^1^O_2_, resulting in a ring-opening process, which leads to the generation of 4‑chloro-2,4-hexadienoic acid. In addition, as depicted in pathway B, the C—Cl bond of 3-CP can attack O_2_^•‒^ via the dechlorination process and produce phenol. The hydroxyl functional group and para-position of the carbon atom in phenol reacts with ^1^O_2_ and leads to the formation of p-benzoquinone. Next, the C = O bond in p-benzoquinone is further attacked by ^1^O_2_ to generate the ring-opening product 4-hydroxyhexa-2,4-dienoic acid. As a result, the ring-opening products of 3-CP with O_2_^•‒^ and ^1^O_2_ lead to decreased inhibition from 100% to 72.8%. In the second stage, the toxicity reduction rate is only 3.2% because the oxidized product 3-chlor-1-buten through alkene-cooixdation possesses high toxicity compared to the above-mentioned intermediates. Finally, the short chain organics will be mineralized to H_2_O, CO_2_ and Cl^−^, with a continuous decrease in the toxicity to 43.3%.

### 3.5 The real application of photoelectrochemical coactivation for oxygen and PMS in the treatment of salinity wastewater

The discharge of wastewater containing high salinity and high organic content results in detrimental effects on aquatic life, water potability and even agriculture [1,2]. In addition, the degradation efficiency for wastewater treatment processes mediated with HO^•^ is limited by side reactions, especially under high salinity conditions [1,3]. However, the ^1^O_2_-dominated photoelectrochemical process exhibits great potential for the treatment of highly saline wastewater in which inorganic ions can serve as natural electrolytes. Thus, real water samples from fine chemical plant effluent, coal slurry gasification effluent, integrated industrial plant effluent and textile dying factory effluent were selected for investigating the degradation efficiency of the novel photoelectrochemical method in this work. The detailed basic characteristics of these wastewaters are summarized in Table S9. Dissolved organic carbon (DOC) and dissolved organic nitrogen (DON) are essential parameters for assessing water quality since DOC can directly reflect the dissolved organic materials in the water matrix and DON can indirectly indicate the toxicity and eutrophication of the water body [36,42,43]. As shown in Fig. 5c, the DOC removal for the four kinds of saline wastewater is 64.7%, 78.5%, 86.2%, and 85.1% after 4 h, respectively. The corresponding DON removal is 71.6%, 88.7%, 92.4%, and 96.2%, respectively. Obviously, the removal efficiency for DON is higher than that for DOC, probably because the nitrogen-containing electron-rich groups (amino, amide, pyrrole, etc.) in wastewater are more easily attacked by ^1^O_2_. These findings confirm that the SA-Fe_0.36_CN/PE(PMS/O_2_) system can maintain the efficient removal of organic pollutants under hypersaline conditions and avoid the adverse effects of eutrophication caused by plant effluent. Coal wastewater and complex industrial wastewater are two typical high salinity wastewaters that represent wastewater obtained from different industrial sources. Therefore, we chose coal wastewater and complex industrial wastewater to further investigate the removal of COD and energy consumption. As depicted in Fig. 5d, half COD removal was accomplished in 60 min for coal wastewater and complex industrial wastewater. Moreover, high COD removal (> 90%) was achieved within 180 min in the PE (PMS/O_2_) system with SA-Fe_0.36_CN, confirming that ^1^O_2_ is efficient for the purification of high-salinity wastewater containing electron-rich contaminants. In addition, the specific energy consumption (SEC) was evaluated based on removing 1 g COD from the wastewater sample (kW·h· *g*^−^^1^) by using Eq. 7:

(7) $$\mathrm{SEC}=\frac{U\times I\times \Delta t}{{\mathrm{COD}}_{0}\times V_{0}-{\mathrm{COD}}_{t}\times V_{t}}$$

where *COD*_*0*_ and *COD*_*t*_ are the chemical oxygen demands for organic compounds at times 0 and t, respectively. *V*_*0*_ and *V*_*t*_ are the volumes of the reactor (30 mL), while U, I, and ∆t are the average voltages (2.8 V), applied current (0.02 A), and photoelectrolysis time (h), respectively.

The SEC is determined to be 0.0011 kW·h·*g*^−^^1^ and 0.0021 kW·h·*g*^−^^1^ when the COD removal for coal wastewater and complex industrial wastewater reaches a value of ∼90% after 180 min, respectively (Fig. 5d). In addition, the energy consumption in terms of the electrical energy per order was investigated (Fig. S26). Electrical energies on the order of 2.05 kW·h·*m*^−^^3^ ·order^−1^ and 6.44 kW·h·*m*^−^^3^·order^−1^ are required for 90% COD removal for coal wastewater and complex industrial wastewater, respectively. The energy consumption remains relatively low during the whole degradation process, indicating that the designed electrodes maintain a higher stability even under high ionic strength conditions.

## 4 Conclusion

High salinity wastewater treatment has dominated industrial fields over the past century, although increasing challenges have resulted in a shift in demand to more green and economic approaches. ^1^O_2_ as an electrophilic oxidant shows unique superiority for the purification of saline wastewater containing electron-rich contaminants. Herein, we propose a new strategy for boosting ^1^O_2_ generation via coactivation of oxygen and peroxymonosulfate in photoelectrochemical processes. A series of atomic Fe-doped graphitic carbon nitrides are designed with electron-defective Fe centers and electron-rich N vacancies. The electron transfer regime can be regulated via the formation of a Fe-N bridge, in which the produced intermediate (HSO_4__—_O···Fe−Nvs···O—O) as a chemical channel facilitates directional electron transfer during the coactivation of O_2_ and PMS. The optimized ^1^O_2_ generation rate reaches 39.4 μmol *L*^−^^1^ *s*^−^^1^, resulting in the efficient degradation of various electron-rich phenolic pollutants. Almost all dissolved organic carbon (DOC) and dissolved organic nitrogen (DON) is removed in fine chemical plant effluent, coal slurry gasification effluent, integrated industrial plant effluent, and textile dying factory effluent. Electrical energies on the order of 2.05 kW·h·*m*^−^^3^·order^−1^ and 6.44 kW·h·*m*^−^^3^·order^−1^ are required for 90% COD removal for coal, and complex industrial wastewater was selected as a reference. This work highlights that ^1^O_2_-mediated oxidation processes can be desirable treatment options for selective environmental remediation under high salinity conditions.

## Declaration of competing interest

The authors declare that they have no conflicts of interest in this work.

## Appendix Supplementary materials

Image, application 1
