# Supplementary material for: Boosting singlet oxygen generation for salinity wastewater treatment through co-activation of oxygen and peroxymonosulfate in photoelectrochemical process
Source: Fundam Res. 2022 Dec 23;5(1):165–73. doi: 10.1016/j.fmre.2022.12.007 (PMC11955032; doi:10.1016/j.fmre.2022.12.007)
Supplement: Supplementary file 1 [file mmc1.docx]

**Supporting Information**

***for***

**Boosting Singlet Oxygen Generation for Salinity Wastewater Treatment through Co-activation of Oxygen and Peroxymonosulfate in Photoelectrochemical process**

Qianqian Yang^a,1^, Zhiyuan Feng^a,1^, Yanbo Zhou^b^ Hongying Zhao^a^*, Guohua Zhao^a^

^a^ Shanghai Key Lab of Chemical Assessment and Sustainability, Key Laboratory of Yangtze River Water Environment, School of Chemical Science and Engineering, Tongji University, 1239 Siping Road, Shanghai 200092, China

^b^ Key Laboratory of Coal Gasification and Energy Chemical Engineering of Ministry of Education, East China University of Science and Technology, Meilong Road 130,

Shanghai, 200092, P.R. China

* Corresponding authors Tel.: +86-(0)21-65988570-8436;

Fax: +86-(0)21-65982287

* E-mail addresses: [hyzhao@tongji.edu.cn](mailto:hyzhao@tongji.edu.cn)

^1^ These authors contributed equally to this work and are regarded as co-first authors.

**Contents**

**[Experimental Section](#_Toc9476)** [1](#_Toc9476)

**[Text S1](#_Toc3421)**[. Semi-quantify the concentration of](#_Toc3421) ^[1](#_Toc3421)^[O](#_Toc3421)_[2](#_Toc3421)_ [by normalized double integration of the EPR 6](#_Toc3421)

**[Text S2](#_Toc31272)** [Quantitative determination of](#_Toc31272) ^[1](#_Toc31272)^[O](#_Toc31272)_[2](#_Toc31272)_ [formation by MB-ADPA method 7](#_Toc31272)

**[Figure S1](#_Toc2737)**[. XRD patterns of CN, SA-Fe](#_Toc2737)_[0.08](#_Toc2737)_[CN, SA-Fe](#_Toc2737)_[0.36](#_Toc2737)_[CN, NP-Fe](#_Toc2737)_[0.08](#_Toc2737)_[CN, NP-Fe](#_Toc2737)_[0.36](#_Toc2737)_[CN 9](#_Toc2737)

**[Figure S2](#_Toc15112)**[. Overall XPS spectra for SA-Fe](#_Toc15112)_[0.36](#_Toc15112)_[CN. 10](#_Toc15112)

**[Figure S3](#_Toc4124)**[. XPS spectra for each elements in SA-Fe](#_Toc4124)_[0.36](#_Toc4124)_[CN. 10](#_Toc4124)

**[Figure S4](#_Toc31429)**[. FTIR of CN, SA-FeCN and NP-FeCN electrodes 12](#_Toc31429)

**[Figure S5](#_Toc26765)**[. TEM patterns of CN, SA-FeCN and NP-FeCN electrodes. 13](#_Toc26765)

**[Figure S6](#_Toc5019)**[. UV-vis diffuse reflectance spectra of CN, SA-FeCN and NP-FeCN 14](#_Toc5019)

**[Figure S7](#_Toc31793)**[. The steady-state photoluminescence spectra (PL) of CN, SA-FeCN and NP-FeCN 15](#_Toc31793)

**[Figure S8](#_Toc28765)**[. Photocurrent responses of CN, SA-FeCN and NP-FeCN. 16](#_Toc28765)

**[Figure S9](#_Toc2177)**[. electrochemical impedance spectroscopy of CN, SA-FeCN and NP-FeCN 17](#_Toc2177)

**[Figure S10](#_Toc9928)**[. Linear fitting curve of SA-Fe](#_Toc9928)_[0.36](#_Toc9928)_[CN, and reference materials derived from the corresponding Fe K-edge XANES spectra. 18](#_Toc9928)

**[Figure S11](#_Toc2504)**[. WT-EXAFS for Fe](#_Toc2504)_[2](#_Toc2504)_[O](#_Toc2504)_[3](#_Toc2504)_[. 19](#_Toc2504)

**[Figure S12](#_Toc4474)**[. H](#_Toc4474)_[2](#_Toc4474)_[O](#_Toc4474)_[2](#_Toc4474)_ [concentration in electrocatalysis (E), photocatalysis (P) and photoelectrocatalysis (PE) proccess with SA-Fe](#_Toc4474)_[0.36](#_Toc4474)_[CN, respectively. 20](#_Toc4474)

**[Figure S13](#_Toc27072)**[. Linear sweep voltammetry curves for SA-Fe](#_Toc27072)_[0.36](#_Toc27072)_[CN in electrocatalysis and photoelectrocatalysis process. 21](#_Toc27072)

**[Figure S14](#_Toc20110)**[. EPR spectra of BMPO-O](#_Toc20110)_[2](#_Toc20110)_^[•-](#_Toc20110)^ [adduct for SA-Fe](#_Toc20110)_[0.36](#_Toc20110)_[CN in photoelectrochemical coactivation of oxygen and peroxymonosulfate (PMS) process. 22](#_Toc20110)

**[Figure S15](#_Toc25656)**[. Normalised double integration peak area of TEMP-](#_Toc25656)^[1](#_Toc25656)^[O](#_Toc25656)_[2](#_Toc25656)_ [for SA-Fe](#_Toc25656)_[0.36](#_Toc25656)_[CN in photoelectrochemical PMS and oxygen co-activation process. 23](#_Toc25656)

**[Figure S16](#_Toc28715)**[. Normalised double integration peak area of TEMP-](#_Toc28715)^[1](#_Toc28715)^[O](#_Toc28715)_[2](#_Toc28715)_ [for SA-Fe](#_Toc28715)_[0.36](#_Toc28715)_[CN under N](#_Toc28715)_[2](#_Toc28715)_ [atmosphere. 24](#_Toc28715)

**[Figure S17](#_Toc23019)**[. Degradation of FFA in the photolysis of MB in photoelectrochemical PMS and oxygen co-activation process. 25](#_Toc23019)

**[Figure S18](#_Toc22160)**[. Linear relationship between](#_Toc22160) ^[1](#_Toc22160)^[O](#_Toc22160)_[2](#_Toc22160)_ [formation and logarithm of ADPA degradation in photoelectrochemical PMS and oxygen co-activation process. 26](#_Toc22160)

**[Figure S19](#_Toc19171)**[. a) Top view and side view of the charge density difference of NP-Fe](#_Toc19171)_[0.36](#_Toc19171)_[CN; b) PMS adsorption on the surface of NP-Fe](#_Toc19171)_[0.36](#_Toc19171)_[CN; c) O](#_Toc19171)_[2](#_Toc19171)_ [adsorption on the surface of NP-Fe](#_Toc19171)_[0.36](#_Toc19171)_[CN; d) The electron transfer orientation and number of co-activation PMS and O](#_Toc19171)_[2](#_Toc19171)_ [process 27](#_Toc19171)

**[Figure S20](#_Toc10071)**[. The TOC removal efficiency of 4-NP, 3-CP, 2,4-DCP, 2,4,5-TCP with SA-Fe0](#_Toc10071)_[.36](#_Toc10071)_[CN under 2h degradation. 28](#_Toc10071)

**[Figure S21](#_Toc15648)**[. The effect of pH value on 3-CP degradation efficiency in SA-Fe](#_Toc15648)_[0.36](#_Toc15648)_[CN under O](#_Toc15648)_[2](#_Toc15648)_ [atmosphere. 29](#_Toc15648)

**[Figure S22](#_Toc13324)**[. The effect of different ions on degradation efficiency of 3-CP with NP-Fe](#_Toc13324)_[0.36](#_Toc13324)_[CN 30](#_Toc13324)

**[Figure S23](#_Toc14686)**[. The reactivity inhibition of 3-CP degradation on SA-Fe](#_Toc14686)_[0.36](#_Toc14686)_[CN in the presence of various scavengers 31](#_Toc14686)

**[Figure S24](#_Toc29264)**[. XRD patterns of fresh and used SA-Fe](#_Toc29264)_[0.36](#_Toc29264)_[CN in the degradation of actual salinity wastewater. 33](#_Toc29264)

**[Figure S25](#_Toc5939)**[. Removal efficiency of PMSO by SA-Fe](#_Toc5939)_[0.36](#_Toc5939)_[CN in coactivation of oxygen and PMS in the photoelectrochemical process 34](#_Toc5939)

**[Figure S26](#_Toc31249)**[. Electrical energy consumption values for coal wastewater and complex industrial wastewater. 35](#_Toc31249)

**[Table S1](#_Toc23251)**[. EXAFS fitting parameters at the Fe K-edge for SA-Fe](#_Toc23251)_[0.36](#_Toc23251)_[CN and FePc. 36](#_Toc23251)

**[Table S2](#_Toc25259)**[. Element analysis of the different samples. 37](#_Toc25259)

**[Table S3](#_Toc14907)**[. The comparison of the rates of](#_Toc14907) ^[1](#_Toc14907)^[O](#_Toc14907)_[2](#_Toc14907)_ [production with various activation process. 38](#_Toc14907)

**[Table S4](#_Toc24045)**[. Bader charge and charge transfer of forming the different active sites. 39](#_Toc24045)

**[Table S5](#_Toc19619)**[. The adsorption energy of PMS and O](#_Toc19619)_[2](#_Toc19619)_ [on single atom Fe and Nvs. 40](#_Toc19619)

**[Table S6](#_Toc10320)**[. Charge transfer and the dissociation energy of PMS in the process of co-activation of PMS and O](#_Toc10320)_[2](#_Toc10320)_ [within SA-Fe](#_Toc10320)_[0.36](#_Toc10320)_[CN/NP-Fe](#_Toc10320)_[0.36](#_Toc10320)_[CN. 41](#_Toc10320)

**[Table S7](#_Toc15953)**[. The molecular structures of the four investigated organic pollutants. 42](#_Toc15953)

**[Table S8](#_Toc24549)**[. Intermediate products in 3-CP degradation process determined by gas chromatography-time-of-flight mass spectrometry (GC-MS) analysis. 43](#_Toc24549)

**[Table S9](#_Toc29775)**[. The basic parameter for the high salinity wastewater. 44](#_Toc29775)

**[References](#_Toc8141)** [45](#_Toc8141)

#

# 1. Experimental Section

## 1.1 Synthesis of SA-FeCN and NP-FeCN electrode

First, the CP was firstly treated in diluted HCl, deionized water and acetone for 5 minutes, then was immersed in concentrated H_2_SO_4_ (98%) for 2 hours. After that it was washed with deionized water and dried at 60 ℃ for 12 h [1]. The obtained clean CP was cut into small pieces of 2cm×3cm for the further preparation of series SA-FeCN and NP-FeCN catalysts. 40 mg of above obtained catalyst and 10 mg of iodine were dissolved into 50 ml acetone and stirred under ultrasonic treatment for 0.5 hour [2]. Then, the CP was impregnated in the mixture containing catalysts for electrodeposition treatment for 20 min, which was carried out with the applied potential of 15V. Finally, the deposited SA-FeCN/CP or NP-FeCN/CP were dried in room temperature and for further activation in vacuum oven at 120℃ for 2 h.

## 1.2 Electrochemical Measurement

Firstly, added 10 mg of catalyst powder into a mixture solution consisting of water, alcohol, Nafion (5 wt%, AlfaAesar) with the volume of 1 mL, 1 mL and 0.25 mL, respectively. Subsequently, 12 uL of the homogeneous mixture solution obtained by ultrasonic treatment for 1 h was extracted and dropped onto a glassy carbon disk electrode (0.283 cm^2^). After overnight drying at room temperature, the prepared working electrode was performed on the linear sweep voltammetry (LSV) test, which was conducted on the computer-controlled CHI 760e electrochemical station (Chenhua Instrument Co. Ltd., China). The three-electrode system equipped with the modified glassy carbon disk working electrode, reference electrode of KCl-saturated Hg/Hg_2_Cl_2_ and counter electrode of platinum wire was operated in the electrolyte of Na_2_SO_4_ [3]. All potentials reported in our work were referenced to a reversible hydrogen electrode (RHE). The LSV experiments were measured in 0.5 M Na_2_SO_4_ between 0 and -1.0 V vs RHE at a scan rate of 5 mV s^-1^. Electrochemical impedance spectra (EIS) data were collected with frequencies ranging from 10^4^ to 10^-2^ Hz at the open circuit potential of 5 mV. All the electrochemical tests were performed at room temperature.

## 1.3 DFT computational details

Density functional theory (DFT) calculations were performed with the Vienna ab initio Simulation Program (VASP) package was used throughout the calculation [4].The projector-augmented wave (PAW) method [5] was used with a plane wave cutoff energy of 400 eV. The Perdew-Burke-Ernzerhof exchange-correlation function was applied in the calculations [6]. A Gaussian smearing with a width of 0.05 was used with the combination of spin polarization throughout the calculations. The electronic energy of the supercell was converged to 10^-6^ eV, with the force on each relaxed atom converged to 0.02 eV/Å in the ionic relaxation calculations.

The g-C_3_N_4_ support was constructed with the p(2×1) supercell was constructed, and the bottom half layer was fixed during structural optimizations. Gamma-centered k-point mesh of (2×2×1) was used because of the large size of the surface modules. A vacuum layer of 15 Å was added to the z direction.

## 1.4 Toxicity tests

The toxicity of 3-Chlorophenol (3-CP) and its intermediate by-products produced during its degradation was assessed by measuring the effect on the luminescence of marine bacterial, as the emitted luminescence of bacterium was related to cell activity [7]. Bacterial luminescence was measured using a Microtox® Model 500 Analyzer (Modern Water Inc.; United Kingdom) with the software MicrotoxOmni. The bacteria used in this method was the strain Vibrio fischeri NRRL B-11177.

Ten concentration series in three parallels and 10 controls were arranged in a microplate and the test was repeated three times. The relative light units (LU) of Vibrio fischeri were determined after 15 min and 12 hours exposure to the toxicant. The toxicity of 3-CP and its intermediates is expressed as the inhibition rate, which is calculated by equation S1:

$I(\%)=(1-\frac{LU(t)}{LU(0)})\times100$ S1

where LU(t) is the intensity of luminescence emitted by bacteria after a t = 15 min or t = 12 hours of contact with the toxicant, LU(0) is the initial intensity of luminescence

emitted by bacteria before the addition of sample.

However, time and the action of environmental conditions also decrease the

luminescence. Errors compensate need to be considered due to these factors by taking

into account the variability of the luminescence R(t) in a control solution as corrected

term, which is calculated by equation S2:

$\text{R(t)= }\frac{LU(t)}{LU0(0)}$ S2

Where LU0(0) is the initial intensity of luminescence emitted by bacteria before

the addition of the control solution (Milli Q water and NaCl).

Then the corrected inhibition rate (Ic) intrinsically attributable to the sample toxicity is calculated using equation S3:

$Ic(\%)=(1-\frac{LU(t)}{R(t)\times LU(0)})\times100$ S3

# Text S1. Semi-quantify the concentration of ^1^O_2_ by normalized double integration of the EPR

The quantification of ^1^O_2_ based on EPR spectra is to detect the number of unpaired electrons in the ^1^O_2_. In EPR experiment, the normalized double integration peak area of the EPR spectra is proportional to the number of unpaired electrons. Therefore, the semi-quantify of ^1^O_2_ can be transformed into the measurement of the normalized double integration peak area for TEMP-^1^O_2_ [8].

Hence, we calculated normalized double integration peak area of TEMP-^1^O_2_ under O_2_ and N_2_ atmosphere in the MATLAB. As shown in Figure S10 and Figure S11, the normalized double integration peak area of TEMP-^1^O_2_ under O_2_ and N_2_ atmosphere were 867.6 and 511.3, respectively.

# Text S2 Quantitative determination of ^1^O_2_ formation by MB-ADPA method

The photolysis of MB was used as an authentic source of ^1^O_2_. The formation rate of ^1^O_2_ in the photolysis of MB was determined by FFA (k^1^O_2 ,FFA_ = 1.2 × 10^8^ M^-1^ s^-1^) [9]. Typical reaction solutions contained 10 μM MB and 100 μM FFA in 50 mM phosphate buffer at pH 7. The liquid phase was 95% D_2_O and 5% H_2_O to increase the steady-state concentration of ^1^O_2_ ([^1^O_2_]_ss_). Assuming the physical quenching of ^1^O_2_ by FFA can be ignored, the steady-state concentration of ^1^O_2_ may be calculated by the following equations:

$\frac{d[FFA]}{dt}=-k_{{}^{1}O_{2},FFA}[{}^{1}O_{2}]_{ss}[FFA]$ S4

$[{}^{1}O_{2}]_{ss}=\frac{\ln([FFA]_{0}/[FFA]_{t})}{dt k_{{}^{1}O_{2},FFA}}$ S5

Accordingly, [^1^O_2_]_ss_ was calculated to be 1.8 × 10^-4^ μM (Figure S10). The formation rate of ^1^O_2_ in the photolysis of MB was calculated by the following equation:

$R_{{}^{1}O_{2},form}=[{}^{1}O_{2}]_{ss}(k_{d}+k_{{}^{1}O_{2},FFA}[FFA])$ S6

where *k_d_* = 2.7×10^4^ s^-1^ is the physical quenching rate constant by solvent (D_2_O/H_2_O = 0.95/0.05, *k_d,H2O_* = 2.7×10^4^ s^-1^ and *k_d,D2O_* = 2.7×10^4^ s^-1^) [10, 11]. For example, the R_1O2 ,form_ was determined to be 7.02 µM s^-1^ in the photolysis of MB. Therefore, the concentration of ^1^O_2_ generated at 10 s, 20 s , 30 s , 40 s , 50 s were 70.2 µM, 140.4 µM, 210.6 µM, 280.8 µM, 351 µM .

Due to FFA can react with PMS, it is not possible to use FFA as probe reagent for ^1^O_2_. The water soluble anthracene derivative salt, anthracene-9,10-dipropionic acid disodium salt (ADPA), was used to trap ^1^O_2_ in PMS system for the presence of ADPA had no significant effect on PMS decomposition and the yield of ADPAO_2_ formation [10]. When produced in the presence of ADPA, ^1^O_2_ can be consumed by three pathways: physical quenching by solvent (k_d_, S7) or ADPA (k_q_, S8), and chemical reaction with ADPA (k_r_, S9).

 S7

 S8

 S9

The loss of ADPA is expressed by:

$\frac{d[ADPA]}{dt}=-k_{r}[{}^{1}O_{2}]_{ss}[ADPA]$ S10

where [^1^O_2_]_ss_ can be calculated from R_1O2, form_ (t) at time t,

$[{}^{1}O_{2}]_{ss}=\frac{R_{{}^{1}O_{2},form}(t)}{k_{d}+k_{q}[ADPA]+k_{r}[ADPA]}$ S11

Substitution of S11 into S10 yields,

$\frac{d[ADPA]}{dt}=-k_{r}[ADPA]\frac{R_{{}^{1}O_{2},form}(t)}{k_{d}+k_{q}[ADPA]+k_{r}[ADPA]}$ S12

So,

$\ln\frac{[ADPA]_{t}}{[ADPA]_{0}}=-\frac{k_{r}}{k_{d}+k_{q}[ADPA]+k_{r}[ADPA]}\int_{0}^{t} R_{{}^{1}O_{2},form}(t)$ S13

$\ln\frac{[ADPA]_{t}}{[ADPA]_{0}}=-\frac{k_{r}}{k_{d}+k_{q}[ADPA]+k_{r}[ADPA]}[{}^{1}O_{2}]_{form}$ S14

where [^1^O_2_]_form_ is the total ^1^O_2_ generated. Figure S11 showed a linear relationship between the formation of ^1^O_2_ and the degradation of ADPA in the photolysis of MB. Therefore, the formation of ^1^O_2_ in PMS processes can be calculated by the degradation of ADPA [10].

# Figure S1. XRD patterns of CN, SA-Fe_0.08_CN, SA-Fe_0.36_CN, NP-Fe_0.08_CN, NP-Fe_0.36_CN

The XRD patterns of CN, SA-Fe_0.08_CN, SA-Fe_0.36_CN, NP-Fe_0.08_CN and NP-Fe_0.36_CN was shown in Figure S1. Only the in-planar ordering of tri-s-triazine units (100) and layer stacking (002) were detected on SA-Fe_0.36_CN electrode, no Fe species were formed according to the XRD patterns. Besides, the diffraction patterns of NP-Fe_0.36_CN displayed three characteristic diffraction peaks at 44.7^o^, 65.2^o^ assigned to the (111) diffraction plane of Fe(0) [12]. However, no Fe species peak were appeared in NP-Fe_0.08_CN XRD patterns, which may attributed to the low loading of Fe precursor.


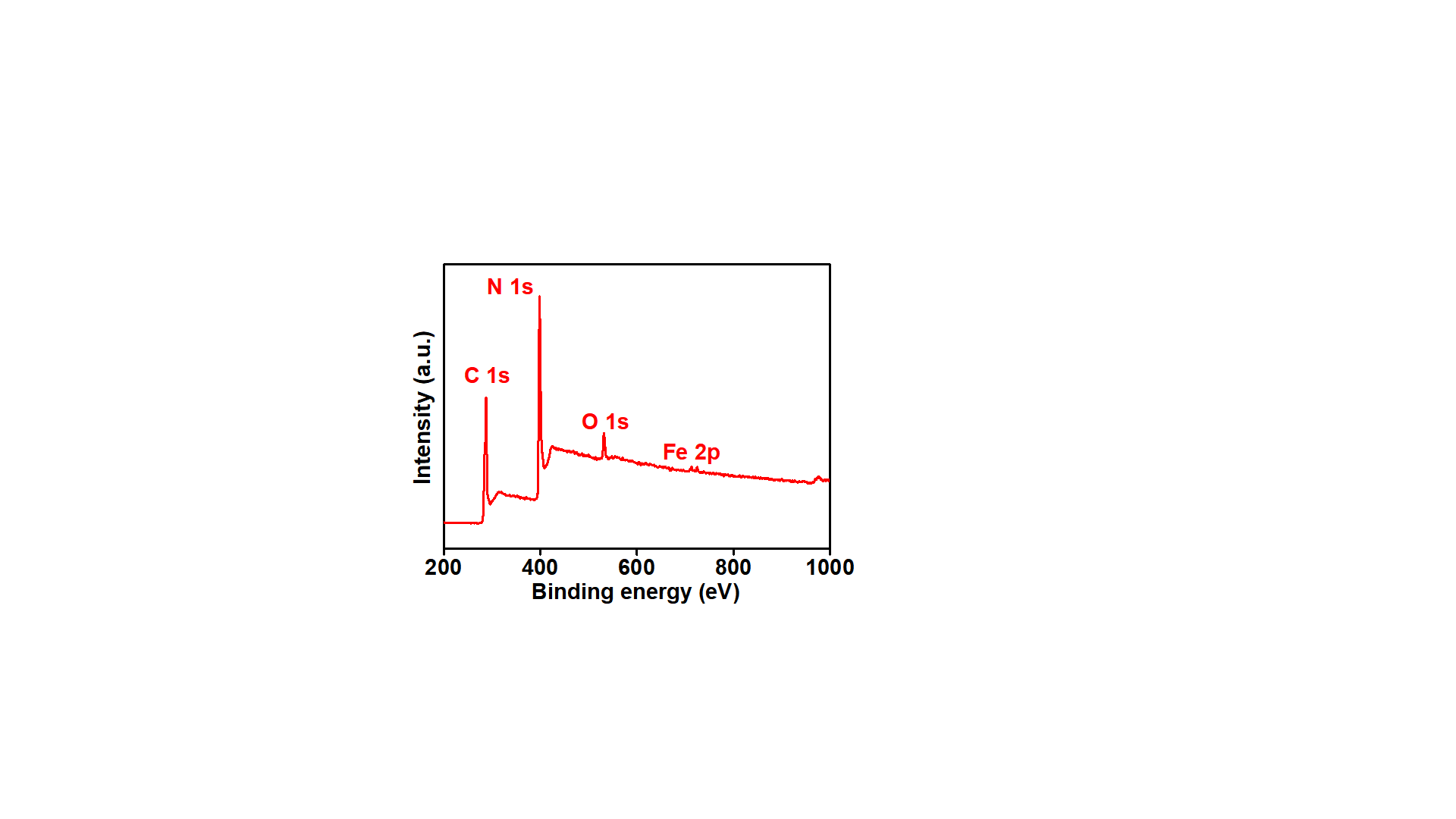


**Figure S2.** Overall XPS spectra for SA-Fe_0.36_CN.


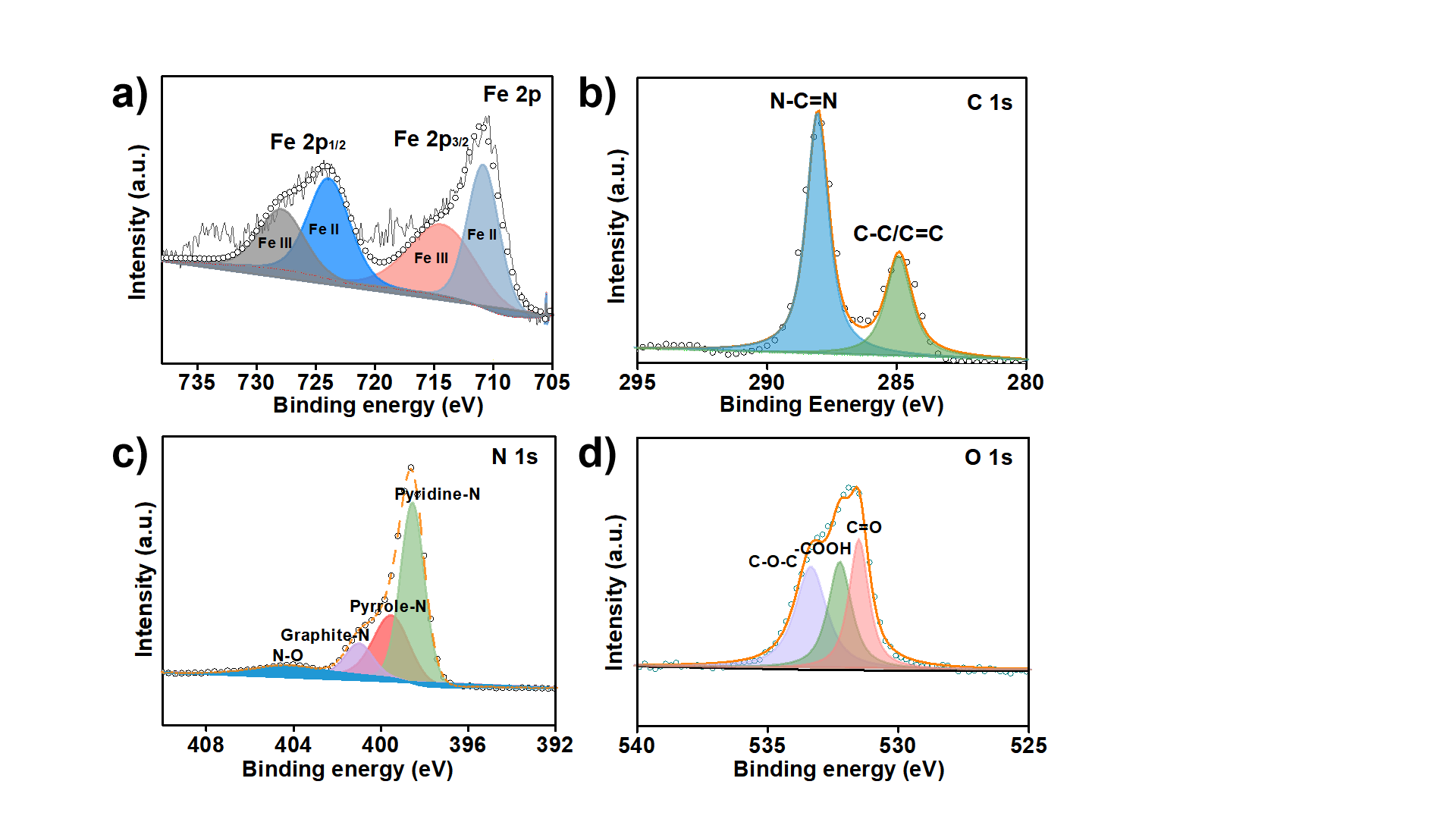


**Figure S3.** XPS spectra for SA-Fe_0.36_CN. a) Fe 2p spectra. b) C 1s spectra. c) N 1s spectra. d) O 1s spectra.

The overall XPS spectra for SA-Fe_0.36_CN were present in Figure S3. The carbon, oxygen, nitrogen and iron element were identified in SA-Fe_0.36_CN. In the Fe 2p spectra for SA-Fe_0.36_CN (Figure S3 a), the two peaks at the binding energy of 723.9 and 710.5 eV are assigned to the characteristic 2p_1/2_ and 2p_3/2_ peaks for Fe^II^ species in Fe-N, and the two peaks at 728.4 and 714.9 eV are attributed to the featured 2p_1/2_ and 2p_3/2_ peaks for the Fe^III^ species in Fe-N, respectively. Besides, as shown in Figure S3 b, the C1s spectrum shows two distinguished peaks centered at 284.8 and 288.1 eV. The former peak is typically ascribed to sp^2^ C-C/C=C bonds, and the latter one is identified as sp-bonded carbon in N-containing aromatic rings (N-C=N). For the N 1s spectrum of SA-Fe_0.36_CN (Figure S3 c), four deconvoluted peaks at the binding energy of 398.5, 399.7, 400.9, and 404.5 eV can be ascribed to the formation of pyridinic-N, pyrrole-N, graphitic-N and free N-O groups, respectively. Both the pyridinic-N and graphitic-N species in SA-Fe_0.36_CN could provide more available photoelectrochemical active sites, facilitating the photoelectrocatalytic activation of O_2_ and PMS. In addition, pyridinic-N could coordinate with Fe species to form stable FeN_4_ structure. Besides, the high-resolution XPS spectra of O 1s band show the presence of C-O-C (533.6 eV), -COOH (532.4 eV) and C=O (531.6 eV) groups that were beneficial to the 2e^-^ ORR process (Figure S3 d).

.

# Figure S4. FTIR of CN, SA-Fe_0.08_CN, SA-Fe_0.36_CN, NP-Fe_0.08_CN, NP-Fe_0.36_CN electrodes

The traditional peak at 1640 cm^-1^ and 1240 cm^-1^ correspond to the stretching vibration of C=N and C-N bonds were obtained for pure CN [13]. The normal vibration of the triazine structure corresponds to the peak at the 808 cm^-1^. Obviously, the introduction of iron species no matter whether atomic sites or nanoparticles didn’t change the skeletal structure of CN.


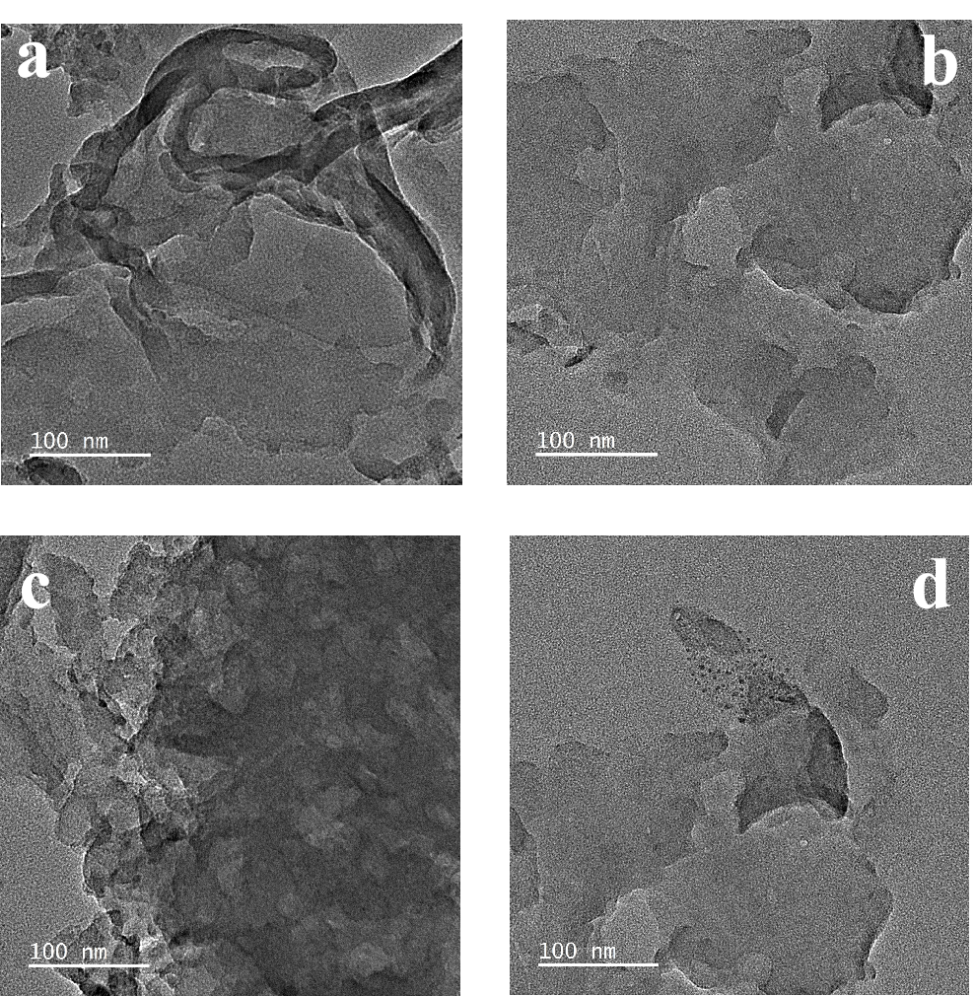


# Figure S5. TEM patterns of (a)SA-Fe_0.08_CN, (b) SA-Fe_0.36_CN, (c) NP-Fe_0.08_CN and (d)NP-Fe_0.36_CN electrodes.

As shown in Figure S5a and S5b, no iron nanoparticles existed in SA-Fe_0.08_CN and SA-Fe_0.36_CN electrodes. However, iron nanoparticles were evidently appeared on the surface of NP-Fe_0.08_CN (Figure S5c) and NP-Fe_0.36_CN (Figure S5d) electrode, which keep the consistent experimental results of XRD patterns.

# Figure S6. UV-vis diffuse reflectance spectra of CN, SA-Fe_0.08_CN, SA-Fe_0.36_CN, NP-Fe_0.08_CN, NP-Fe_0.36_CN

The optical properties were evaluated by ultraviolet-visible (UV-vis) absorption spectra (Figure S6). After anchoring Fe single atoms, the absorption edge of CN exhibits an obvious red-shift and enhance the capacity of light absorption. However, SA-Fe_0.36_CN shows the best light absorption ability. This suggests that solar light absorption was improved by introducing the single atom Fe sites and N vacancies.

# Figure S7. The steady-state photoluminescence spectra (PL) of CN, SA-Fe_0.08_CN, SA-Fe_0.36_CN, NP-Fe_0.08_CN, NP-Fe_0.36_CN

The charge separation efficiency was further clarified by photoluminescence (PL) spectra measurements [14]. As shown in Figure S7, evidently, dramatic steady-state photoluminescence quenching occurs on the SA-Fe_0.36_CN relative to CN and NP-Fe_0.36_CN, implying that the intrinsic radiative recombination is noticeably suppressed by the incorporation of single-atom Fe and adjacent N vacancies.


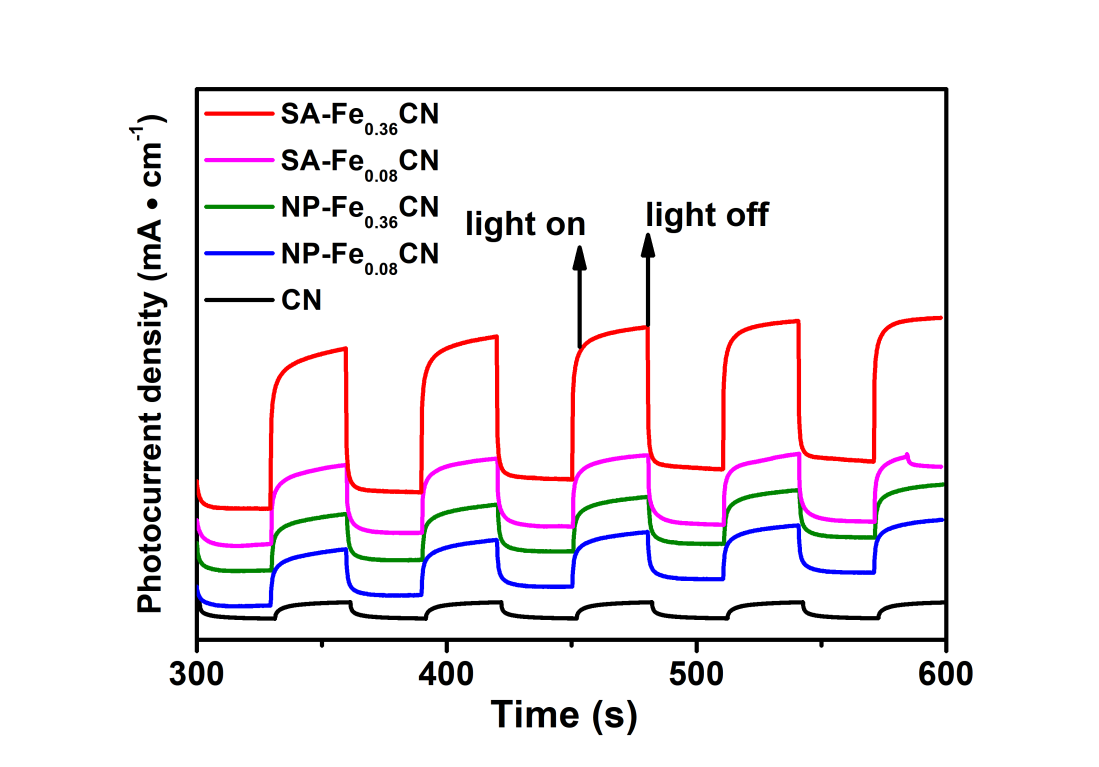


# Figure S8. Photocurrent responses of CN, SA-Fe_0.08_CN, SA-Fe_0.36_CN, NP-Fe_0.08_CN, NP-Fe_0.36_CN in 0.5 M Na_2_SO_4_ aqueous solution under visible light irradiation (λ=420 nm).

Photocurrent responese can comprehensively reflect the photoelectrocatalytic ability of catalysts. The intensity of transient photocurrent response is significantly increased with the introduction of Fe atom and N vacancies. SA-Fe_0.36_CN sample reveals the highest intensity compared with SA-Fe_0.08_CN, NP-Fe_0.08_CN, NP-Fe_0.36_CN and pristine CN. It is suggested that single atom Fe and N vacancies offer a significantly higher enhancement in photoelectrocatalysis ability of SA-Fe_0.36_CN .


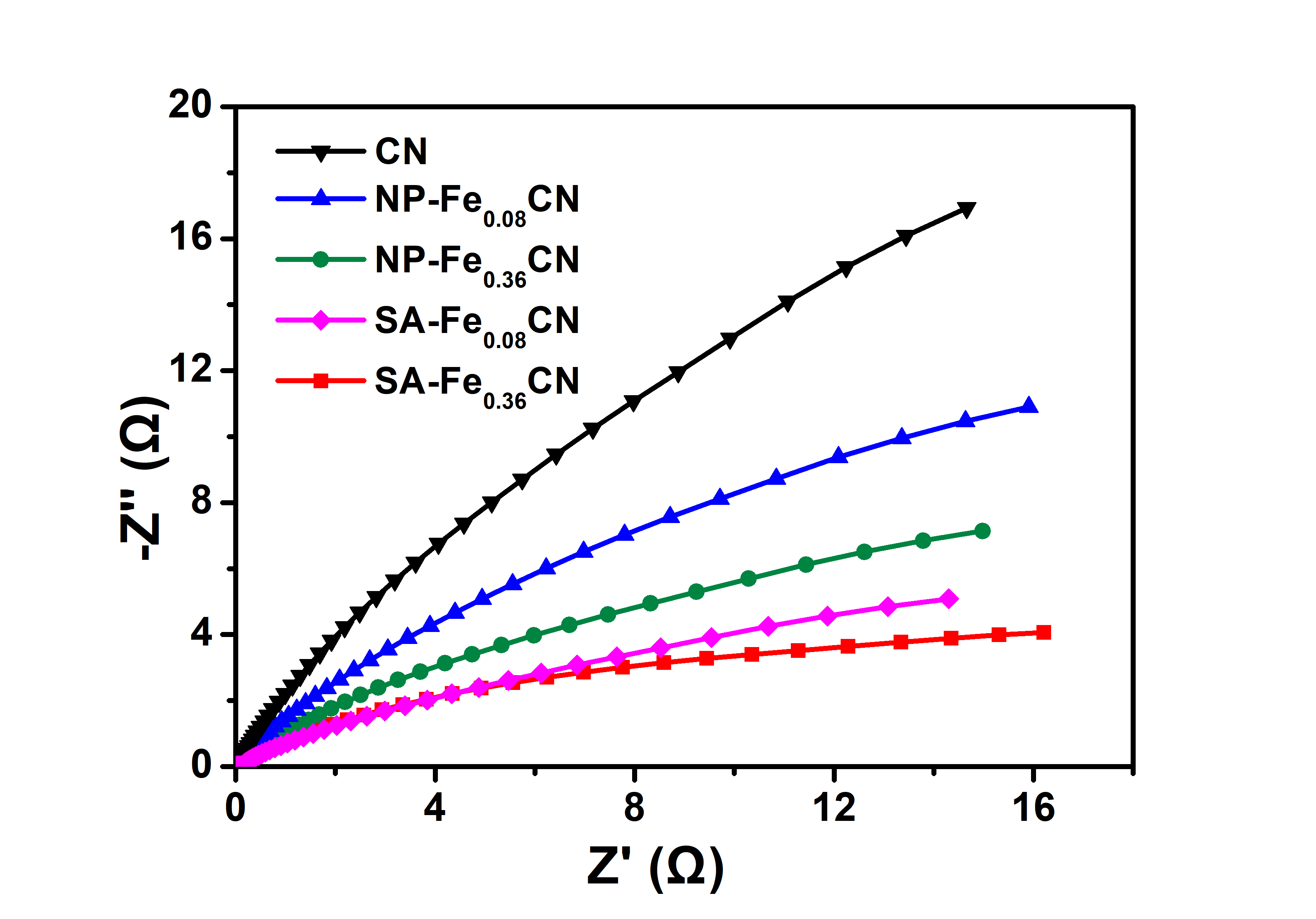


# Figure S9. electrochemical impedance spectroscopy of CN, SA-Fe_0.08_CN, SA-Fe_0.36_CN, NP-Fe_0.08_CN, NP-Fe_0.36_CN.

The internal electron transport capability was characterized by electrochemical impedance spectroscopy [15]. The EIS Nyquist diagram simulates the equivalent electrical circuit well, where Rs and Rt are the electrolyte solution resistance and interfacial charge-transfer resistance, respectively. Based on this model, the electrochemical impedance spectroscopy of SA-Fe_0.36_CN possesses the lowest electrochemical impedance with respect to the CN, NP-Fe_0.08_CN and NP-Fe_0.36_CN, suggesting that interfacial electron transport was enhanced by the modulating of electrons transfer behavior.


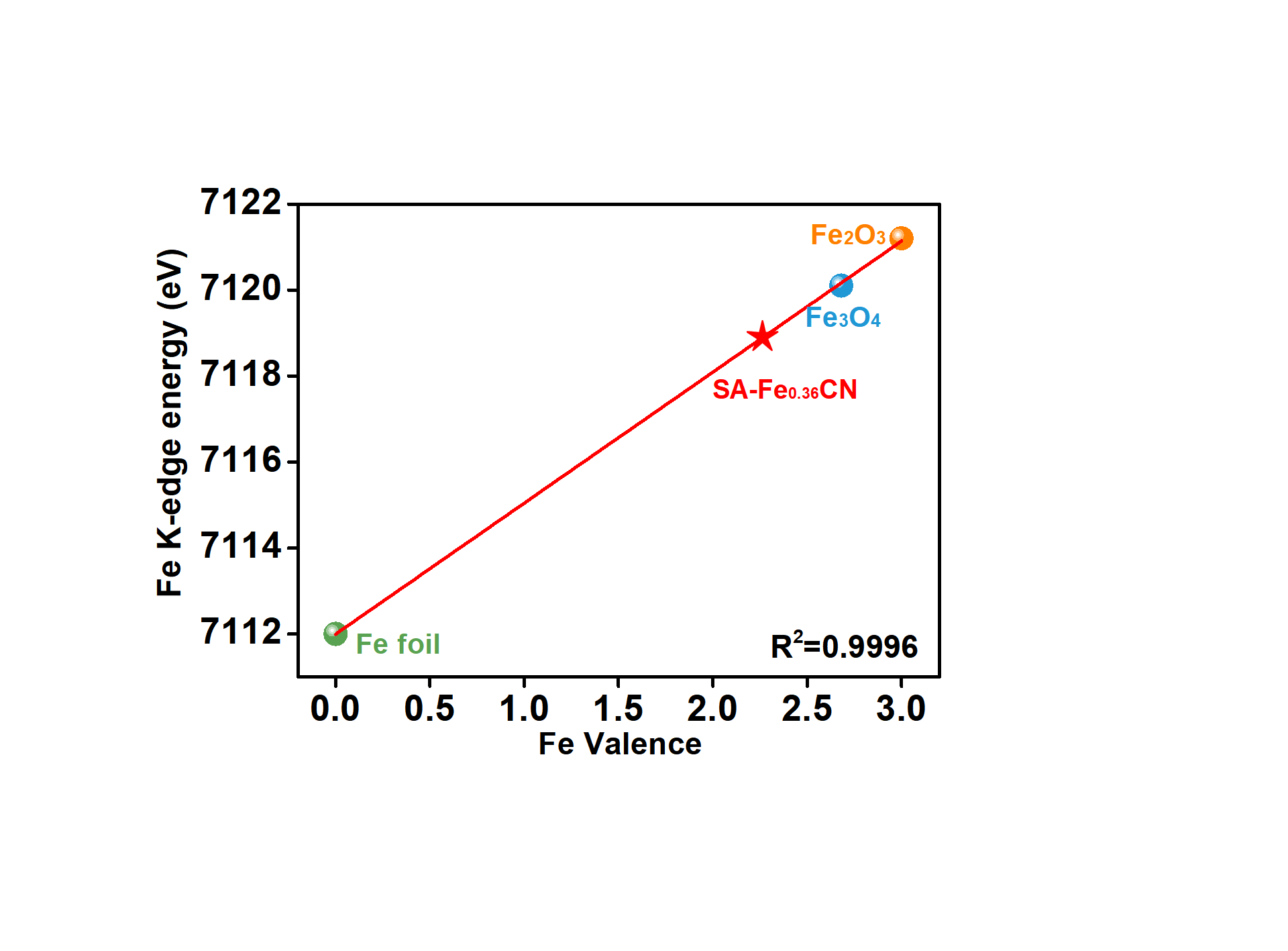


# Figure S10. Linear fitting curve of SA-Fe_0.36_CN, and reference materials derived from the corresponding Fe K-edge XANES spectra.


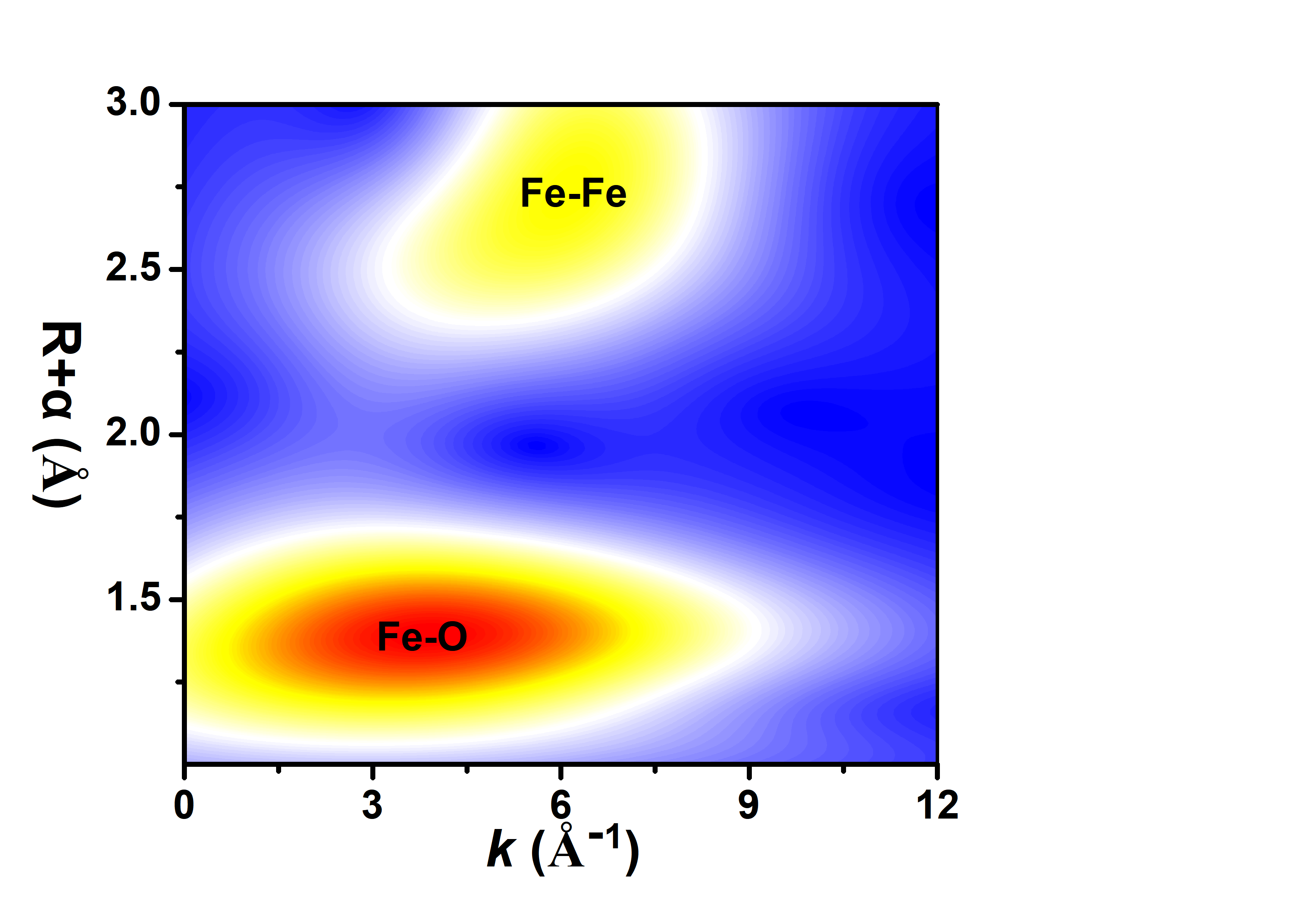


**Figure S11**. WT-EXAFS for Fe_2_O_3_.

# Figure S12. H_2_O_2_ concentration in electrocatalysis (E), photocatalysis (P) and photoelectrocatalysis (PE) proccess with SA-Fe_0.36_CN, respectively.


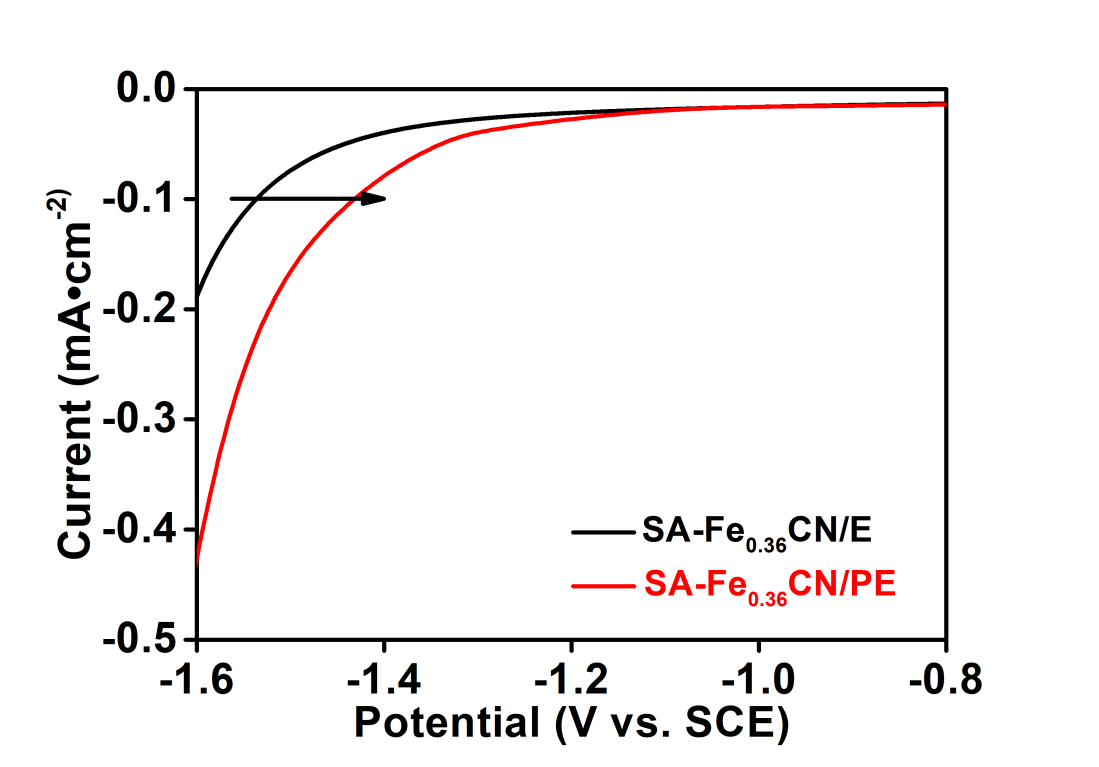


**Figure S13.** Linear sweep voltammetry curves for SA-Fe_0.36_CN in electrocatalysis and photoelectrocatalysis process.

The LSV curves of for SA-Fe_0.36_CN in electrocatalysis and photoelectrocatalysis process were provide in the Figure S13. The current density in the Figure S12 was keep at a constant of -0.1 mA•cm^-2^ for electrocatalysis and photoelectrocatalysis. Therefore, the potential applied for electrocatalysis and photoelectrocatalysis in Figure S12 were -1.53 V and -1.42 V, respectively.


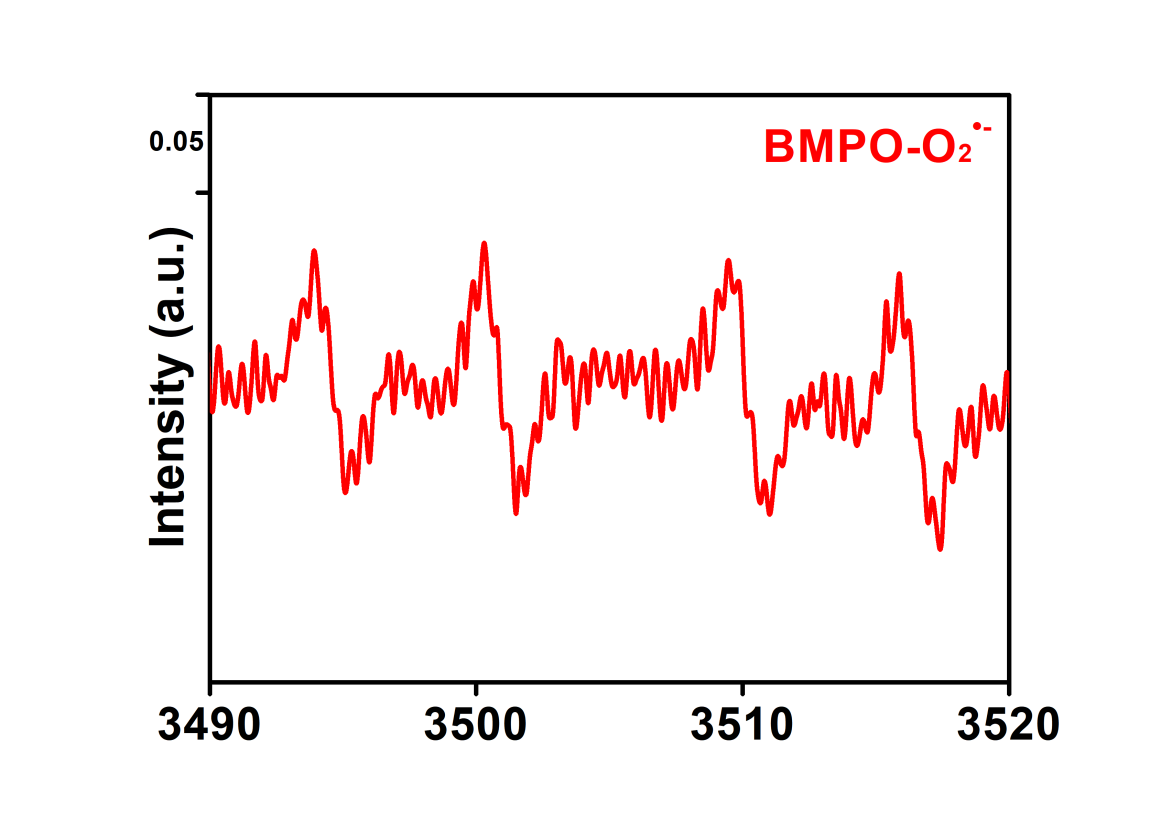


**Figure S14.** EPR spectra of BMPO-O_2_^•-^ adduct for SA-Fe_0.36_CN in photoelectrochemical coactivation of oxygen and peroxymonosulfate (PMS) process.

We qualitative detection the generated O_2_^•-^ during the photoelectrochemical process through using 5-tert-butoxycarbonyl 5-methyl-1-pyrroline N-oxide (BMPO) as trapping agent by EPR method. As shown in Figure S14, the BMPO-O_2_^•-^ spin adduct signal (αN = 12.9 G, αH = 10.3 G, g = 2.0057) is detected in SA-Fe_0.36_CN/PMS+O_2_ reaction system, confirming the formation of O_2_^•-^ during the photoelectrochemical process. However, the intensity of TEMP-^1^O_2_ signal in Figure 2a is 100 times higher than the intensity of BMPO-O_2_^•-^ signal, indicated that O_2_^•-^ as intermediate would be simultaneously converted to ^1^O_2_ during the photoelectrochemical process.

# Figure S15. Normalised double integration peak area of TEMP-^1^O_2_ for SA-Fe_0.36_CN in photoelectrochemical PMS and oxygen co-activation process.

# Figure S16. Normalised double integration peak area of TEMP-^1^O_2_ for SA-Fe_0.36_CN under N_2_ atmosphere.


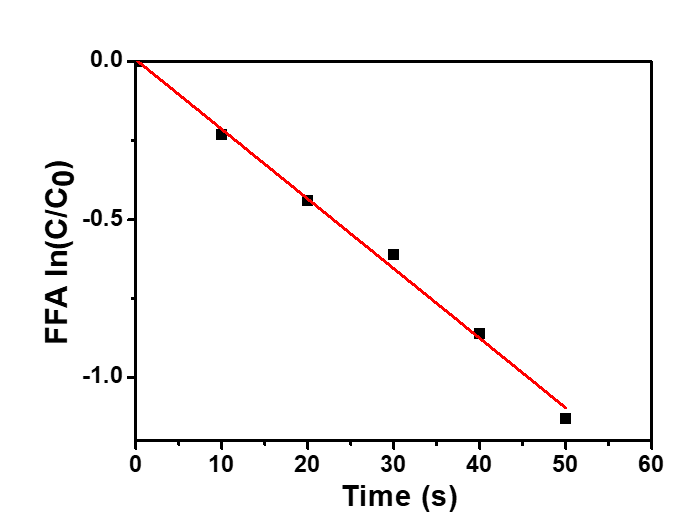


# Figure S17. Degradation of FFA in the photolysis of MB in photoelectrochemical PMS and oxygen co-activation process.


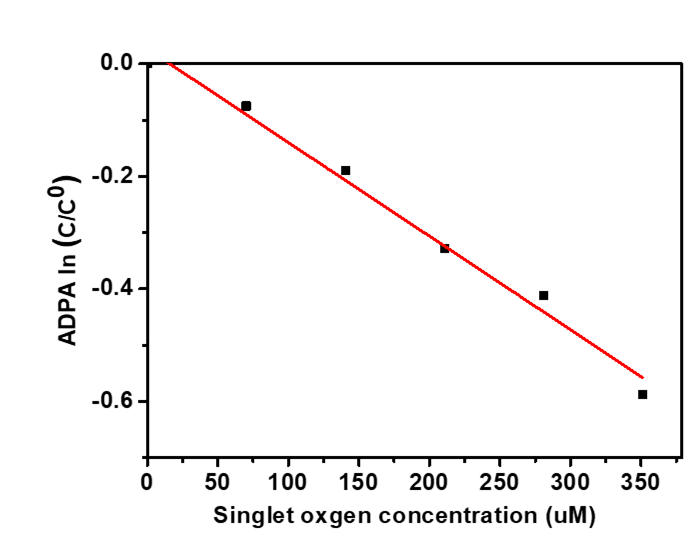


# Figure S18. Linear relationship between ^1^O_2_ formation and logarithm of ADPA degradation in photoelectrochemical PMS and oxygen co-activation process.


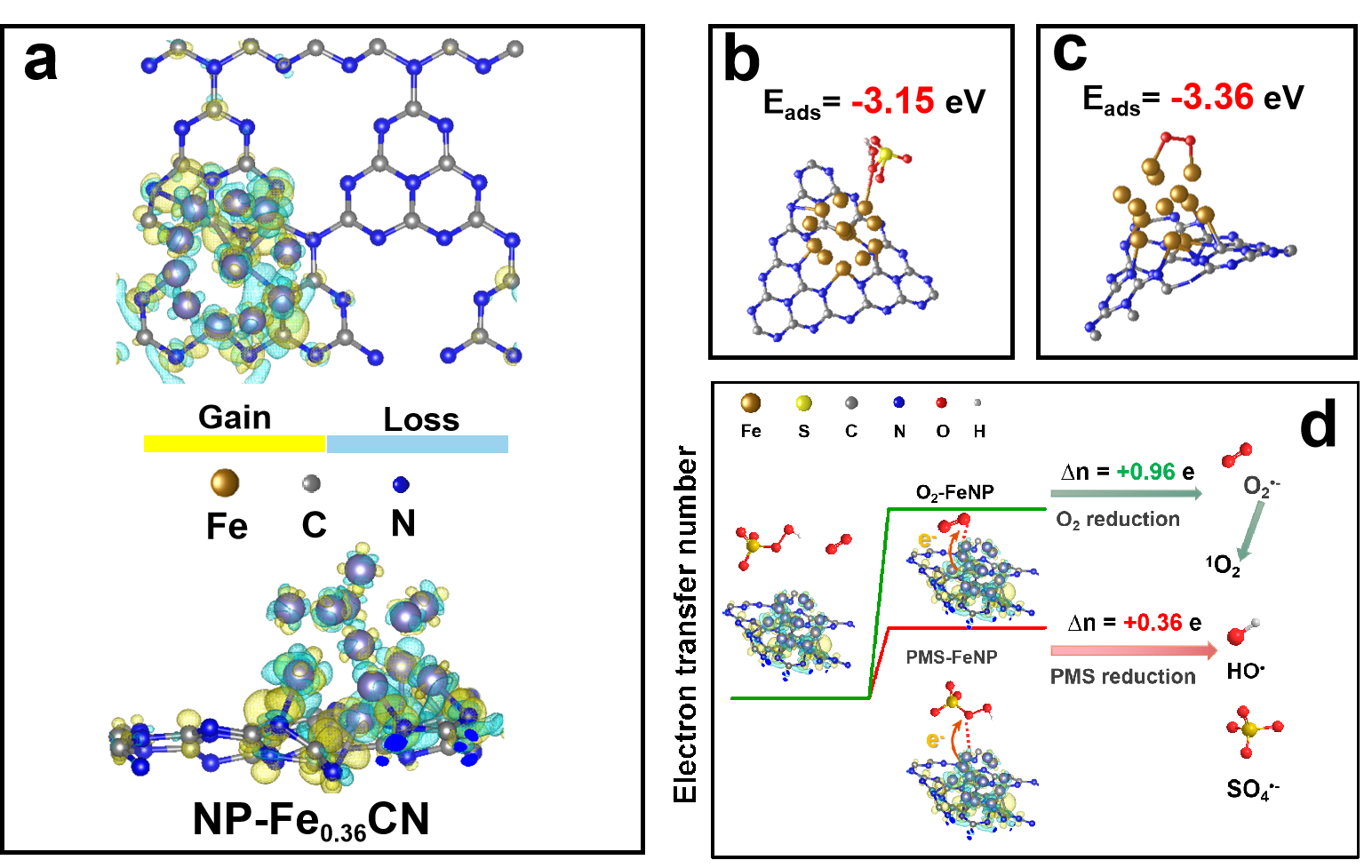


Figure S19. a) Top view and side view of the charge density difference of NP-Fe_0.36_CN; b) PMS adsorption on the surface of NP-Fe_0.36_CN; c) O_2_ adsorption on the surface of NP-Fe_0.36_CN; d) The electron transfer orientation and number of co-activation PMS and O_2_ process.

In Figure S19a, there is no obvious difference in charge distribution of NP-Fe_0.36_CN. Hence, Fe nanoparticles were the unique active site of NP-Fe_0.36_CN. The PMS and O_2_ were both absorbed on the Fe nanoparticles surface, which the adsorption energy were -3.15 eV and -3.36 eV, respectively (Figure S19b and Figure S19c). The charge transfer calculation results indicated that 0.36 e was donated to PMS from Fe nanoparticles. Moreover, the dissociation energy of forming HO^•^and SO_4_^•-^ (E_diss_ =-2.9 eV, Table S6) was lower by forming H^+^ and SO_5_^•-^(E_diss_=4.21 eV, Table S6). Hence, PMS absorbed on NP-Fe_0.36_CN and then tend to be reduced to HO^•^and SO_4_^•-^ . Meanwhile, the Fe nanoparticles can also reduced O_2_ to generate O_2_^•-^ (the electron transform number was 0.96). Then, ^1^O_2_ can be generated through the recombination of O_2_^•-^/O_2_^•-^ and Haber-Weiss reaction.

# Figure S20. The TOC removal efficiency of 4-NP, 3-CP, 2,4-DCP, 2,4,5-TCP with SA-Fe0_.36_CN under 2h degradation.

The TOC removal of 4-NP, 3-CP, 2,4-DCP, 2,4,5-TCP was 20.4%, 57.2% ,35.5% and 26.1%, respectively. Although a large amount of ^1^O_2_ can react with pollutants quickly, the TOC removal efficiency is relatively low. This may be attributed to the insufficient oxidation capacity of ^1^O_2_ to deeply mineralize organic . At the same time, the dechlorination of chlorinated compounds is difficult to be completed by ^1^O_2_ alone, so it also affects the mineralization effect.

# Figure S21. The effect of pH value on 3-CP degradation efficiency in SA-Fe_0.36_CN under O_2_ atmosphere.

As we known, actual wastewater had variable pH values, and thus, it is necessary to determine the effect of pH values on the degradation efficiencies. Obviously, the 3-CP removal rate constant remained as high as 0.57-0.51 min^-1^ in the wide pH range of 3-9, and only 10.5% loss was happened at the pH 9, which indicated that SA-Fe_0.36_CN/PE(PMS/O_2_) was widely applicable in the actual environment.


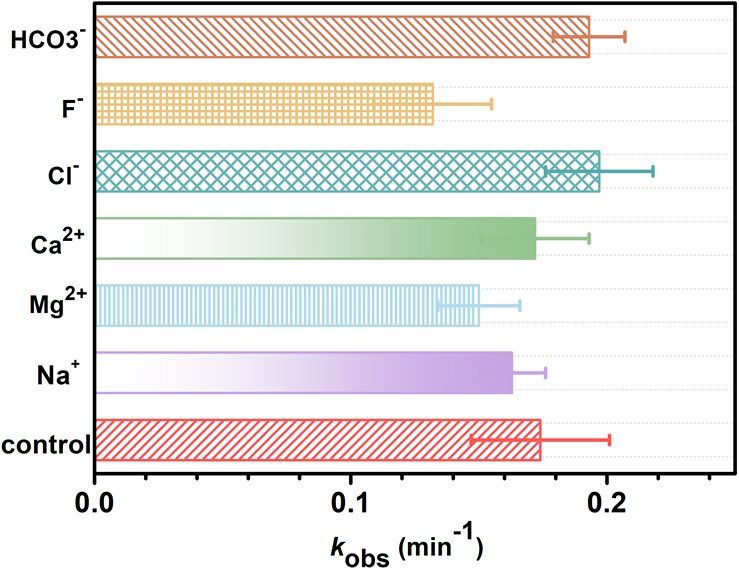


Figure S22. The effect of different ions on degradation efficiency of 3-CP with NP-Fe_0.36_CN.


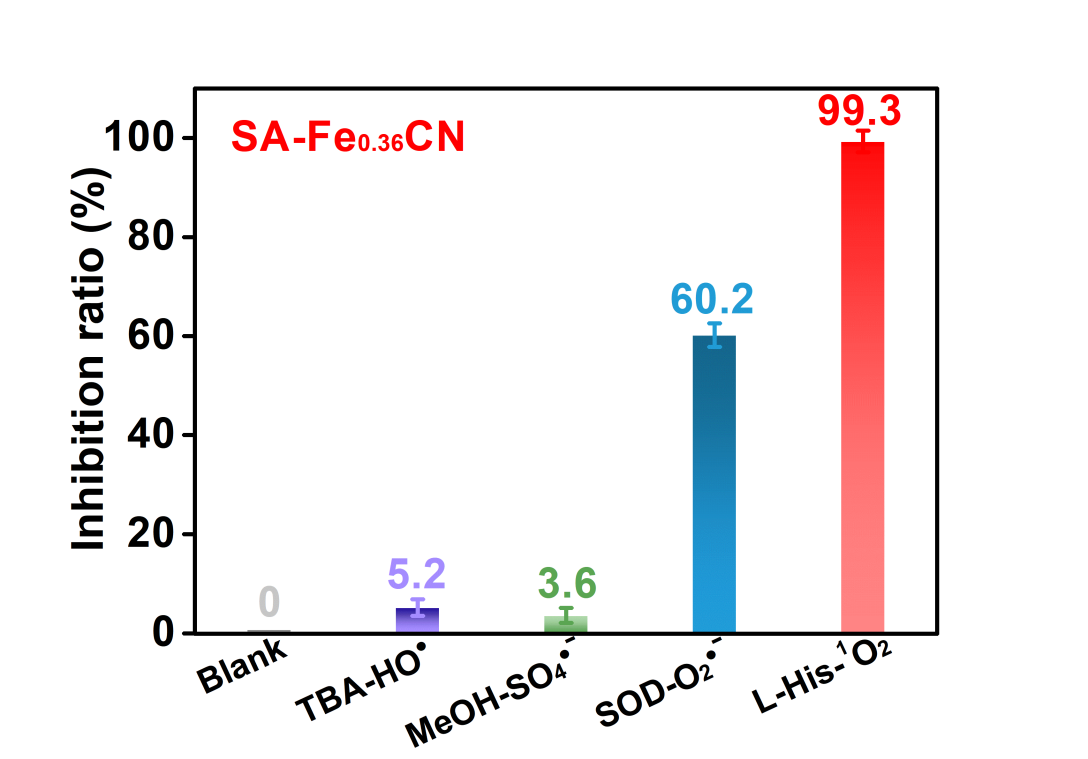


**Figure S23.** The reactivity inhibition of 3-CP degradation on SA-Fe_0.36_CN in the presence of various scavengers. (Scavenger concentration: TBA, MeOH, and L-his was 50 mM, SOD was 50 U• mL^−1^)

The L-histidine (L-his), Tert-butyl alcohol (TBA), superoxide dismutase (SOD) and methanol (MeOH)were choosing as radical scavengers to capture ^1^O_2_, HO^•^, O_2_^•-^ and SO_4_^•-^ in the 3-CP degradation process, respectively. As shown in Figure S23, when TBA and MeOH were added into the reaction solution, only a slight inhibition on 3-CP degradation was observed (5.2% and 3.6%, respectively), implying that the major oxidant species for 3-CP degradation was not HO^•^ and SO_4_^•-^. While, when the SOD was added, the degradation rate was obviously reduced (60.2%). As shown in Eq. 6, O_2_^•-^ was the critical species for ^1^O_2_ generation via oxygen activation pathway, the quenching of O_2_^•-^ will reduce the generation of ^1^O_2_, and then suppress the 3-CP degradation efficiency. In addition, when the L-his was added, almost complete of 3-CP degradation was depressed with a high inhibition of 99.3%. The strong inhibitory effect of L-his evidenced that the generated ^1^O_2_ was major oxidant species for 3-CP removal.


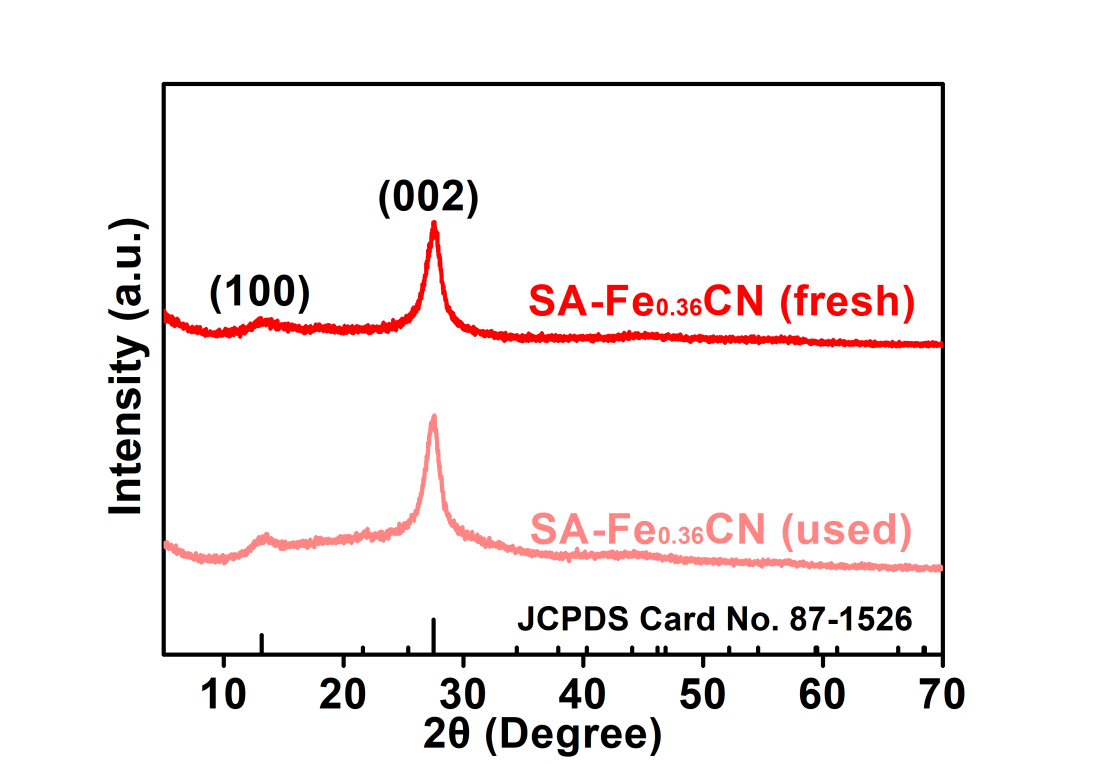


**Figure S24**. XRD patterns of fresh and used SA-Fe_0.36_CN in the degradation of actual salinity wastewater.

The XRD analysis of used SA-Fe_0.36_CN after the degradation of actual salinity wastewater to investigate the stability of the catalyst and the change of its post-catalytic structure and chemical bonding environment. As illustrated in Figure S24, the diffraction patterns of fresh SA-Fe_0.36_CN displayed two characteristic diffraction peaks at 13.2° and 27.6° assigned to the (100) and (002) diffraction plane, respectively, corresponding to the graphic phase with tri-s-triazine unit of g-C_3_N_4_, coincide with the PXRD standard card of g-C_3_N_4_ (JCPDS, Card No. 87-1526), In addition, compared to fresh SA-Fe_0.36_CN, the main diffraction peaks were almost unchanged and no additional peaks were observed for used SA-Fe_0.36_CN. These results indicated that SA-Fe_0.36_CN possessed high stability after the degradation of actual salinity wastewater.


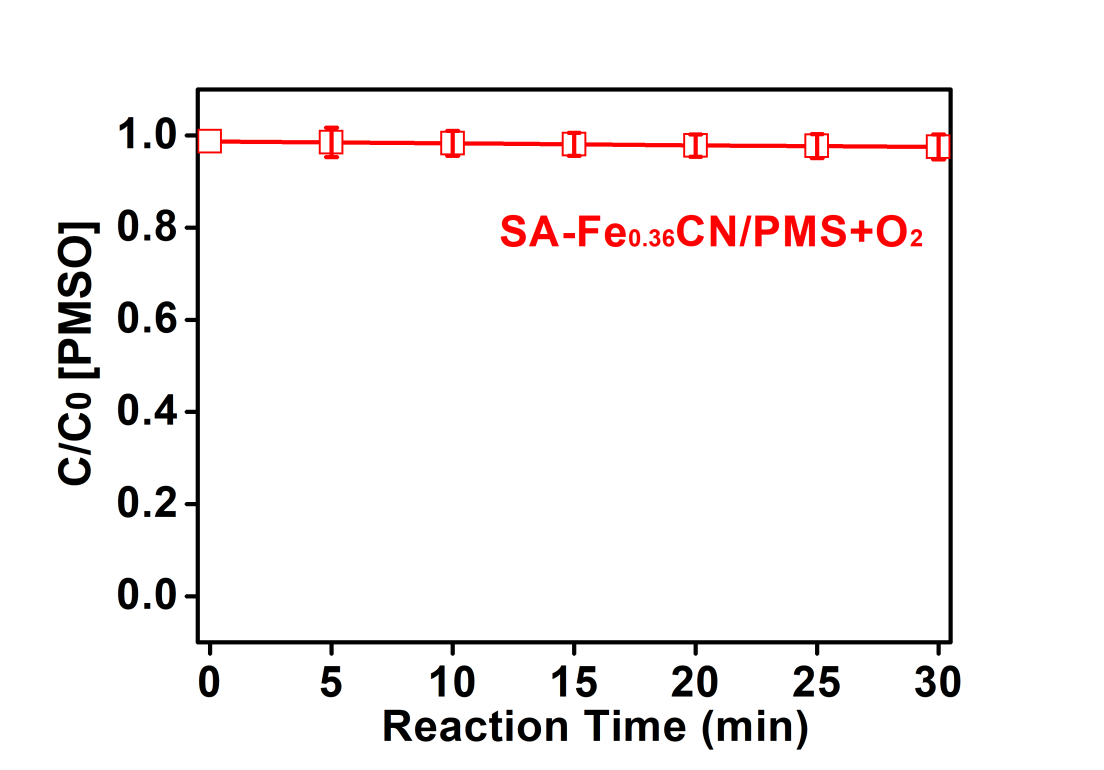


**Figure S25.** Removal efficiency of PMSO by SA-Fe_0.36_CN in coactivation of oxygen and PMS in the photoelectrochemical process. (Conditions: [PMSO] = 10 ppm, other experimental condition were same as the experimental setup provided in supporting information)

The formation of Fe(IV) species was tasted by using methyl phenyl sulfoxide (PMSO) as a chemical probe. As described in the previous literature (*Environ. Sci. Technol.* 2021, 55, 7034−7043), the PMSO could be selectively oxidized to phenyl sulfone (PMSO_2_) by Fe(IV). Thus, it is reasonable to evaluate the formation of Fe(IV) through the degradation performance of PMSO in the SA-Fe_0.36_CN/PMS+O_2_ system. As displayed in Figure S25, the PMSO removal was only 2.5%, indicating that almost no Fe(IV) was produced in SA-Fe_0.36_CN/PMS+O_2_ system.

# Figure S26. Electrical energy consumption values for coal wastewater and complex industrial wastewater.

The major fraction of operating cost in photoelectrocatalysis process is mainly associated with electrical energy per order and it can be defined as the number of kWhr (kilo Watt hour) of electrical energy required to reduce the COD concentration of wastewater by 1st order of magnitude in 1 m^3^ . The electrical energy per order can be calculated by using the equation S15 [3].

$energy per order=\frac{U\times I\times t\times1000}{V\times60\times\ln(\frac{C_{0}}{C_{t}})}(kWh/m^{3}order^{-1})$ S15

Where, U is average voltage (2.8 V), I is applied current (0.02 A), t is the reaction time (in min), V is the volume of reactor (in litre), C and Ct were the COD concentration (in ppm) at initial and time t.

# Table S1. EXAFS fitting parameters at the Fe K-edge for SA-Fe_0.36_CN and FePc.

| Sample | Shell | CNr | R(Å) | σ^2^(A^2^) | R factor |
| --- | --- | --- | --- | --- | --- |
| SA-Fe_0.36_CN | Fe-N | 3.7±0.4 | 1.60±0.03 | 0.012 | 0.013 |
| FePc | Fe-N | 3.9±0.2 | 1.78±0.02 | 0.007 | 0.009 |

CNr: coordination numbers; R: bond distance; σ^2^: Debye-Waller factors; R factor: goodness of fit.

# Table S2. Element analysis of the different samples.

| sample | C/N |
| --- | --- |
| CN | 0.72 |
| SA-Fe_0.36_CN | 0.81 |
| NP-Fe_0.36_CN | 0.75 |

# Table S3. The comparison of the rates of ^1^O_2_ production with various activation process.

| Activation process | Rates of ^1^O_2_  (μ mol L^-1^ s^-1^) | Reaction conditions | References |
| --- | --- | --- | --- |
| PMS+O_2_ activation | **39.4** | [ADPA]= 0.20 mM  [PMS] = 5 mM  Light source: Xenon lamp (λ >420 nm) | **This work** |
| H_2_O_2_ activation | 0.2 | [H_2_O_2_]= 10.0 mM  Catalyst (1 g/L)  Light source: Xenon lamp (λ < 420 nm) | [16] |
| PMS activation | 0.4 | [PMS] = 1.5 mM, Catalyst (0.01 g/L)  [DPBF]= 0.2 mM | [17] |
| PMS activation | 0.5 | [PMS] = 0.5 g/L  Catalyst (0.2 g/L)  [FFA]= 0.85 mM | [18] |
| O_2_ activation | 0.04 | Ethanol (100 ml)  Catalyst (0.25 g/L)  DPBF (0.02 g/L) | [19] |
| O_2_ activation | 2.5 | DBPF (0.2 mM)  Catalyst (0.5 g/L)  Light source: Xenon lamp (λ >420 nm) | [20] |

# Table S4. Bader charge and charge transfer of forming the different active sites.

| Element | Valence  Electron (e) | Bader charge (e) | | Charge transfer (e) | |
| --- | --- | --- | --- | --- | --- |
|  |  | SA-Fe_0.36_CN | NP-Fe_0.36_CN | SA-Fe_0.36_CN | NP-Fe_0.36_CN |
| Fe | 8 | 6.86 | 7.83 | -1.14 | -0.17 |
| C1 | 4 | 4.89 | - | 0.89 | - |
| C2 | 4 | 4.22 | - | 0.22 | - |
| C3 | 4 | 4.20 | - | 0.22 | - |

# Table S5. The adsorption energy of PMS and O_2_ on single atom Fe and Nvs.

| Adsorption site | E_ads, PMS_ (eV) | E_ads, O2_ (eV) |
| --- | --- | --- |
| Fe | -5.29 | -2.74 |
| Nvs | 3.26 | -2.46 |

# Table S6. Charge transfer and the dissociation energy of PMS in the process of co-activation of PMS and O_2_ within SA-Fe_0.36_CN/NP-Fe_0.36_CN.

| Element | Valence  Electron  (e) | Bader charge (e) | | Charge transfer (e) | | dissociation energy of H^+^ and HSO_5_^•-^ (eV) | | dissociation energy of HO^•^ and SO_4_^•-^ (eV) | |
| --- | --- | --- | --- | --- | --- | --- | --- | --- | --- |
|  |  | SA-Fe_0.36_CA | NP-Fe_0.36_CA | SA-Fe_0.36_CA | NP-Fe_0.36_CA | SA-Fe_0.36_CA | NP-Fe_0.36_CA | SA-Fe_0.36_CA | NP-Fe_0.36_CA |
| PMS | 38 | 37.81 | 38.36 | -0.19 | 0.36 | 0.28 | 1.19 | 4.21 | -2.90 |
| O_2_ | 12 | 12.52 | 12.96 | 0.52 | 0.96 | - | - | - | - |

# Table S7. The molecular structures of the four investigated organic pollutants.

| Name | Formula | Molecular structure | Functional substituents |
| --- | --- | --- | --- |
| 3-chlorophenol (3-CP) | C_6_H_5_OCl |  | -OH, -Cl |
| 2,4-Dichlorophenol (2,4-DCP) | C_6_H_4_Cl_2_O |  | -OH, -Cl |
| 2,4,5-Trichlorophenol (2,4,5-TCP) | C_6_H_3_Cl_3_O |  | -OH, -Cl |
| Nitrophenol (4-NP) | C_6_H_5_NO_3_ |  | -OH, -NO_2_ |

# Table S8. Intermediate products in 3-CP degradation process determined by gas chromatography-time-of-flight mass spectrometry (GC-MS) analysis.

| Intermediates | Formula | Molecular structure | m/z |
| --- | --- | --- | --- |
| phenol | C_6_H_6_O |  | 94 |
| 4-chloro-2,4-hexadienoic acid | C_6_H_6_O_2_Cl |  | 145 |
| p-benzoquinone | C_5_H_4_O_2_ |  | 96 |
| 4-hydroxyhexa-2,4-dienoic acid | C_6_H_7_O_2_ |  | 111 |
| 3-chlor-1-buten | C_4_H_7_Cl |  | 90 |
| Fumaric acid | C_4_H_4_O_4_ |  | 116 |
| Malonic acid | C_3_H_4_O_4_ |  | 104 |
| acetic acid | C_2_H_4_O_2_ | **CH_3_COOH** | 60 |

# Table S9. The basic parameter for the high salinity wastewater.

| Parameters | Chemical  wastewater | Coal wastewater | Complex industrial wastewater | Textile dying wastewater |
| --- | --- | --- | --- | --- |
| Total dissolved solids (TDS, mg/L) | 27633 | 117256 | 3450 | 3290 |
| Initial COD value (mg L^-1^) | 5280 | 3700 | 2800 | 5200 |
| Initial DOC value (mg L^-1^) | 4198 | 476 | 87 | 39 |
| Initial DON value (mg L^-1^) | 3906 | 571 | 52 | 3 |

**References**

[1] Z. Zhao, F. Qin, S. Kasiraju, L. Xie, M.K. Alam, S. Chen, D. Wang, Z. Ren, Z. Wang, L.C. Grabow, J. Bao, Vertically aligned MoS_2_/Mo_2_C hybrid nanosheets grown on carbon paper for efficient electrocatalytic hydrogen evolution, ACS Catal. 7 (2017) 7312-7318.

[2] Q. Yang, Z. Feng, M. Liu, J. Zhang, H. Zhao, G. Zhao, A general strategy via photoelectrocatalytic oxygen reduction for generating singlet oxygen with carbon bridged carbon-nitride electrode, Chinese Chem. Lett. 32 (2021) 3393-3397.

[3] X. Shen, F. Xiao, H. Zhao, Y. Chen, C. Fang, R. Xiao, W. Chu, G. Zhao, In situ-formed PdFe nanoalloy and carbon defects in cathode for synergic reduction-oxidation of chlorinated pollutants in electro-Fenton process, Environ. Sci. Technol. 54 (2020) 4564-4572.

[4] G. Kresse, J. Furthmüller, Efficiency of ab-initio total energy calculations for metals and semiconductors using a plane-wave basis set, Comp. Mater. Sci. 6 (1996) 15-50.

[5] J.P. Perdew, K. Burke, M. Ernzerhof, Generalized gradient approximation made simple, Phys. Rev. Lett. 77 (1996) 3865-3868.

[6] P.E. Blöchl, Projector augmented-wave method, Phys. Rev. B 50 (1994) 17953-17979.

[7] M. Liu, Z. Feng, X. Luan, W. Chu, H. Zhao, G. Zhao, Accelerated Fe^2+^ regeneration in an effective electro-Fenton process by boosting internal electron transfer to a nitrogen-conjugated Fe(III) complex, Environ. Sci. Technol. 55 (2021) 6042-6051.

[8] Z. Yang, J. Qian, A. Yu, B. Pan, Singlet oxygen mediated iron-based Fenton-like catalysis under nanoconfinement, Proc. Natl. Acad. Sci. U. S. A, 116 (2019) 6659-6664.

[9] E. Appiani, R. Ossola, D.E. Latch, P.R. Erickson, K. McNeill, Aqueous singlet oxygen reaction kinetics of furfuryl alcohol: effect of temperature, pH, and salt content, Environ. Sci: Proc. Imp. 19 (2017) 507-516.

[10] Y. Yang, G. Banerjee, G.W. Brudvig, J.-H. Kim, J.J. Pignatello, Oxidation of organic compounds in water by unactivated peroxymonosulfate, Environ. Sci. Technol. 52 (2018) 5911-5919.

[11] W.R. Haag, J.r. Hoigne´, E. Gassman, A.M. Braun, Singlet oxygen in surface waters — Part I: Furfuryl alcohol as a trapping agent, Chemosphere 13 (1984) 631-640.

[12] F. Xiao, Z. Wang, J. Fan, T. Majima, H. Zhao, G. Zhao, Selective electrocatalytic reduction of oxygen to hydroxyl radicals via 3-electron pathway with FeCo alloy encapsulated carbon aerogel for fast and complete removing pollutants, Angew Chem. Int. Ed. Engl. 60 (2021) 10375-10383.

[13] L.J. Fang, X.L. Wang, J.J. Zhao, Y.H. Li, Y.L. Wang, X.L. Du, Z.F. He, H.D. Zeng, H.G. Yang, One-step fabrication of porous oxygen-doped g-C_3_N_4_ with feeble nitrogen vacancies for enhanced photocatalytic performance, Chem. Commun. 52 (2016) 14408-14411.

[14] H. Li, F. Li, Z. Wang, Y. Jiao, Y. Liu, P. Wang, X. Zhang, X. Qin, Y. Dai, B. Huang, Fabrication of carbon bridged g-C_3_N_4_ through supramolecular self-assembly for enhanced photocatalytic hydrogen evolution, Appl. Catal. B: Environ. 229 (2018) 114-120.

[15] G. Dong, K. Zhao, L. Zhang, Carbon self-doping induced high electronic conductivity and photoreactivity of g-C_3_N_4_, Chem. Commun. 48 (2012) 6178-6180.

[16] J. Jiang, X. Wang, Y. Liu, Y. Ma, T. Li, Y. Lin, T. Xie, S. Dong, Photo-Fenton degradation of emerging pollutants over Fe-POM nanoparticle/porous and ultrathin g-C_3_N_4_ nanosheet with rich nitrogen defect: Degradation mechanism, pathways, and products toxicity assessment, Appl. Catal. B: Environ. 278 (2020) 119349.

[17] L. Jin, S. You, X. Duan, Y. Yao, J. Yang, Y. Liu, Peroxymonosulfate activation by Fe_3_O_4_-MnO_2_/CNT nanohybrid electroactive filter towards ultrafast micropollutants decontamination: Performance and mechanism, J. Hazard. Mater. 423 (2022) 127111.

[18] P. Gao, X. Tian, Y. Nie, C. Yang, Z. Zhou, Y. Wang, Promoted peroxymonosulfate activation into singlet oxygen over perovskite for ofloxacin degradation by controlling the oxygen defect concentration, Chem. Eng. J. 359 (2019) 828-839.

[19] J. Ding, Z. Dai, F. Tian, B. Zhou, B. Zhao, H. Zhao, Z. Chen, Y. Liu, R. Chen, Generation of defect clusters for ^1^O_2_ production for molecular oxygen activation in photocatalysis, J. Mater. Chem. A. 5 (2017) 23453-23459.

[20] Z. Zhou, Z. Shen, C. Song, M. Li, H. Li, S. Zhan, Boosting the activation of molecular oxygen and the degradation of tetracycline over high loading Ag single atomic catalyst, Water Res. 201 (2021) 117314.
